# Extended Regression Models for Predicting the Pumping Capability and Viscous Dissipation of Two-Dimensional Flows in Single-Screw Extrusion

**DOI:** 10.3390/polym11020334

**Published:** 2019-02-14

**Authors:** Wolfgang Roland, Michael Kommenda, Christian Marschik, Jürgen Miethlinger

**Affiliations:** 1 Institute of Polymer Extrusion and Compounding, Johannes Kepler University Linz, 4040 Linz, Austria; christian.marschik@jku.at (C.M.); juergen.miethlinger@gmail.com (J.M.); 2 Josef Ressel Centre for Symbolic Regression, School of Informatics, Communication and Media, University of Applied Sciences Upper Austria, 4232 Hagenberg, Austria; Michael.Kommenda@fh-hagenberg.at

**Keywords:** polymer processing, modeling and simulation, extrusion, symbolic regression, power-law fluid

## Abstract

Generally, numerical methods are required to model the non-Newtonian flow of polymer melts in single-screw extruders. Existing approximation equations for modeling the throughput–pressure relationship and viscous dissipation are limited in their scope of application, particularly when it comes to special screw designs. Maximum dimensionless throughputs of $\Pi_{V}<2.0$, implying minimum dimensionless pressure gradients $\Pi_{p,z}\geq -0.5$ for low power-law exponents are captured. We present analytical approximation models for predicting the pumping capability and viscous dissipation of metering channels for an extended range of influencing parameters ($\Pi_{p,z}\geq -1.0$, and $t/D_{b}\leq 2.4$) required to model wave- and energy-transfer screws. We first rewrote the governing equations in dimensionless form, identifying three independent influencing parameters: (i) the dimensionless down-channel pressure gradient $\Pi_{p,z}$, (ii) the power-law exponent n, and (iii) the screw-pitch ratio $t/D_{b}$. We then carried out a parametric design study covering an extended range of the dimensionless influencing parameters. Based on this data set, we developed regression models for predicting the dimensionless throughput-pressure relationship and the viscous dissipation. Finally, the accuracy of all three models was proven using an independent data set for evaluation. We demonstrate that our approach provides excellent approximation. Our models allow fast, stable, and accurate prediction of both throughput-pressure behavior and viscous dissipation.

## 1. Introduction

Single-screw extruders are the most important machinery in polymer processing. They are used in continuous extrusion lines to produce finished or semi-finished products (e.g., pipes, sheets, films and profiles) and in recycling. However, single-screw plasticizing units are not limited to continuous processes, but are also used in injection molding and as feeding units in blow molding and thermoforming processes. Hence, a majority of the polymers produced pass a single-screw unit at least once in their life cycle. The melt-conveying zone—also often referred to as the pumping zone—is one of the most crucial functional zones in single-screw extruders. It must convey the melt forward and provide sufficient pressure to pump it through the die. Especially in smooth-bore single-screw extruders, the melt-conveying zone is the rate-limiting zone and must generate most of the pressure needed at the screw tip. In grooved-fed single-screw extruders, however, the solids conveying zone is already capable of building up most of the required pressure, and the following zones are often over ridden. Hence, the melt-conveying zone can be both pressure-generating and pressure-consuming. Further, a significant proportion of the total dissipated energy is generated within the metering zone, which, therefore, has a major influence on the final melt temperature.

### 1.1. Review

The first analysis of the metering zone was published anonymously in 1922 [1] and further improved by Rowell and Finlayson in 1928 [2]. They developed a one-dimensional isothermal Newtonian pumping model using the flat-plate model, a widely accepted approach to describing the flow channel. Assuming the viscosity to be Newtonian and temperature-independent, an analytical model was derived for the melt-conveying behavior where the total flow rate is a linear superposition of drag- and pressure-flows, which can be solved independently. Several further studies have investigated and extended the theory for Newtonian fluids. Carley et al. [3] included the die characteristic and tapered screw channels. However, not only the throughput-pressure relation is of interest, but also the power and torque required to drive the screw. Based on the existing Newtonian flow theory, an equation for the power consumption was therefore presented by Mallouk and McKelvey [4]. The screw drive power is given by the product of the viscous force acting on the barrel surface and its area. The cross-channel flow was first included by Mohr et al. [5]. They derived the down- and cross-channel velocity profiles independently and superposed them to derive the shear imposed on the fluid. In a subsequent study Mohr and Mallouk [6] included the cross-channel flow in the calculation of the power requirement. In order to validate the Newtonian pumping model, McKelvey [7] performed experiments on a single-screw extruder using a viscous Newtonian fluid (corn syrup) and a nearly Newtonian polymer melt.

These early studies of Newtonian fluids gave first insights into the polymer melt flow in single-screw extruders. Nevertheless, for more accurate analyses, the non-Newtonian fluid behavior of polymer melts must be taken into account. Polymer melts generally exhibit shear-thinning—also called pseudo-plastic—fluid behavior. This means that the viscosity decreases with increasing shear rate. Hence, drag and pressure flows are no longer independent of each, which results in a non-linear relationship for the throughput-pressure characteristics. Additionally, down- and cross-channel flows influence each other, albeit not directly but via the shear-rate-dependent viscosity. Taking the non-Newtonian fluid behavior into account significantly increases the complexity of the problem, and the flow equations can no longer be solved analytically, but require numerical techniques. Even for the simplest non-Newtonian case—a one-dimensional, isothermal flow of a power-law fluid without flight effects—no exact, closed-form analytical solution has yet been found [8]. Several authors [8,9,10,11,12] have presented analytical solutions for the Poiseuille-Couette flow of a power-law fluid that require the integration constant to be determined numerically. In principle, all these solutions give similar results. Krüger [10] additionally proposed a representative viscosity approach that uses the linear Newtonian to approximate the non-linear throughput-pressure characteristic. Kroesser and Middleman [11] compared their results with the traditional Newtonian pumping model and the superposition introduced by Krüger, applying the representative viscosity. A general and detailed derivation of the analytical approach for calculating the throughput-pressure characteristic of a one-dimensional flow of a power-law fluid within an infinite channel width was provided by Tadmor and Gogos [12]. For accurate predictions, however, the cross-channel flow cannot be ignored, because it influences the down-channel flow via the shear-rate-dependent viscosity, and vice versa. Hence, the flow rate is also affected by the transverse flow. Addressing this issue, Griffith [13] derived numerical results for isothermal and non-isothermal fully developed two-dimensional flows of power-law fluids. For the non-isothermal case, a constant fluid temperature is assumed for one streamline. Zamodits and Pearson [14] followed a similar approach, reporting results for an isothermal flow, as well as a flow with a superposed steady temperature profile of a power-law fluid for pseudo-plastic and dilatant fluids. Stellar [15] presented an algebraic solution for the two-dimensional flow of a power-law fluid. This algebraic solution, however, requires the integration constants for the velocity profiles to be solved numerically. An alternative approach was introduced by Booy [16], who investigated the effect of the transverse flow on the effective viscosity. Detailed reviews of the traditional melt-conveying models were published by Tadmor and Klein [17] and by Fenner [18].

All the approaches to modeling the non-Newtonian throughput-pressure relationship of polymer melts in single-screw extruders presented above require numerical methods. For practical screw design, however, numerical methods are not always desirable, because they are complex, require high computational effort, and can be time-consuming and unstable. For this reason, Rauwendaal [8,19] introduced correction factors that describe the non-Newtonian behavior. Applying these correction factors allows the Newtonian throughput-pressure relationship to be used, while avoiding complex numerical calculations. Note that these correction factors only hold for pressure-generating zones with the helix angle in the range from 15° to 25°. Potente [20,21] followed a similar approach: First, an approximation was presented of the one-dimensional flow of power-law fluids for pressure-generating and pressure-reducing melt-conveying zones that is valid for a range of the dimensionless throughput rate of $0.55\leq \Pi_{V}\leq 1.45$ [20]. Then, the model was improved with regard to the effect of the transverse flow on the throughput-pressure relationship [21]. It was shown that the improved two-dimensional model covers the numerical values in the range $0.55\leq \Pi_{V}\leq 1.0$ for the dimensionless throughput and in the range $0{}^{\circ}\leq \varphi_{b}\leq 17.65{}^{\circ}$ for the helix angle. A further improvement of Potente’s model was developed by Effen [22], who extended the range of the dimensionless influencing parameters. Effen’s model is valid for the range $0.1\leq \Pi_{V}\leq 2.0$ for the dimensionless throughput, 0.2≤n≤1.0 for the power-law exponent, and $0.8\leq t/D_{b}\leq 2.0$ for the screw-pitch ratio. His model, however, distinguishes between eight different regions with different values for the model coefficients. A detailed summary of Potente’s and Effen’s models was given by White and Potente [23]. The models described above are all linear or piecewise-linear approximations of the throughput-pressure relationship. A generalized two-dimensional model that describes both pressure-generating and pressure-consuming melt-conveying zones ($0.0\leq \Pi_{V}\leq 2.0$) was presented by Pachner et al. [24]. Additionally, it considers wide ranges of the screw pitch ratio ($0.75\leq t/D_{b}\leq 2.0$) and the power-law exponent (0.2≤n≤0.9). Pachner et al. avoided the traditional formulation that superposes the drag- and pressure-flows and developed a closed analytical approximation equation for the throughput-pressure relationship, applying symbolic regression based on genetic programming. Spalding and Campbell [25,26] provided a correction factor for the rotational flow—drag flow—that considers the deviation of the drag flow of power-law fluids in finite channels compared to the drag flow for a Newtonian fluid. Their approach, however, does not include a shape factor for the pressure-flow. Kim and Kwon [27] introduced a total shape factor to include the influence of the flights on the flow rate. This total shape factor gives the ratio between the flow rate of the three-dimensional flow to that by the two-dimensional. Nevertheless, an analytical approximation is only given for Newtonian fluid. Marschik et al. [28,29,30] presented a three-dimensional metering model for polymer melts, taking into account the rate-limiting effect of the screw flights on the flow rate. By means of experiments [31] and three-dimensional simulations of wave-screws [32] the validity and practicability of the regression models [24,29] in capturing the melt-conveying zone of single-screw extruders was demonstrated.

For practical screw design, not only the isothermal throughput-pressure relationship, but also viscous dissipation, power-consumption, and melt-temperature development, are of great significance. Mallouk and McKelvey [4] demonstrated the power calculation for Newtonian fluids. Mohr and Mallouk [6] extended the analysis to include the transverse flow. McKelvey [33] introduced the calculation of the adiabatic melt temperature development for a temperature-dependent Newtonian fluid leading to a logarithmic melt temperature increase. Tadmor and Klein [17] summarized this work, pointing out that the real extrusion process lies between the adiabatic and isothermal conditions, and that the adiabatic case defines the maximum melt temperature to be expected. Including the non-Newtonian fluid behavior of polymer melts, Potente [21] developed approximation equations for the drive power of melt-conveying zones, considering one- and two-dimensional flows. These models apply in the range $0.55\leq \Pi_{V}\leq 1.25$ for the dimensionless throughput. Potente and Obermann [34] further improved these models, performing 2.5D finite-element (FE) simulations. Application of these equations for non-isothermal throughput-pressure calculations that take into account constant Brinkmann numbers was shown by White and Potente [23]. However, the power consumption is not directly responsible for the melt-temperature increase. Rather, the screw-drive power is a combination of pressure build-up work and viscous dissipation, where the latter is the main source of the melt-temperature increase in a melt-conveying channel. Campbell et al. [35] investigated the viscous dissipation of a Newtonian fluid for both barrel- and screw-rotation theory. Using a representative viscosity approach, Rauwendaal [8] developed various methods for predicting the melt-temperature increase. In his approaches, the pressure and temperature development are coupled via a temperature-dependent viscosity. With this representative viscosity, the viscous dissipation is approximated simply by pure drag flow. Thus, the additional dissipation caused by the pressure flow, as well as the coupling between drag- and pressure flows, and the transverse flow are ignored. Derezinski [36,37,38] presented numerical solutions for the axial melt-temperature developments of power-law and Carreau-Yasuda viscosities. In his work, heat conduction through the barrel wall was taken into account by heat transfer coefficients and a fixed barrel-wall temperature. However, a representative shear rate was used for calculating the viscosity. Note that all these models for calculating the axial melt-temperature development are based on the lumped energy equation, and therefore predict a bulk melt temperature. Using a representative shear rate is insufficient for an accurate prediction of the viscous dissipation—the contributions of the pressure flow, the effect of the non-Newtonian fluid behavior, and the coupling effect of pressure-, drag-, and transverse flows via the shear-rate-dependent viscosity cannot be ignored. To address this matter, previous studies derived numerical results [39] and presented generalized symbolic regression models [40] of one- and two-dimensional flows of power-law fluids. These models cover a wide range of processing conditions ($0.0\leq \Pi_{V}\leq 2.0$) of polymeric materials (0.2≤n≤1.0) and screw geometries ($0.5\leq t/D_{b}\leq 2.0$). Further, they enable fast, simple, accurate, and stable prediction of viscous dissipation in the melt-conveying zone.

The novelty of recent work [24,29,40] by our research group is that analytical approximation equations are generated using symbolic regression based on genetic programming. In contrast to classical regression analysis, in symbolic regression based on genetic programming, the structure of the model does not have to be predefined. This enables the identification of unknown non-linear relationships. Koza [41] was the first who described the principles of symbolic regression in detail. Schmidt and Lipson [42] demonstrated that genetic programming is suitable for discovering analytical relationships that underlie physical phenomena in nature by automatically searching motion-tracking data. In modeling the non-linearity of electric machines, Bramerdorfer et al. [43] applied symbolic regression using genetic programming to data derived by finite-element simulations. Symbolic regression was shown to achieve the highest accuracies among various other modeling strategies. It is not limited to simulation data, but can also be applied to experimental data, as shown by Kronberger et al. [44] in modeling wet tribological systems; they first carried out extensive experimental tests and then applied symbolic regression. However, data-based modeling using experimental data may require data preprocessing. Symbolic regression is also not limited to technical contexts, but can be applied in any field, for instance, physics, biology, medicine, and economy, as shown in [45] for macro-economic time-series modeling.

### 1.2. Overview

In this work, we present extended symbolic regression models for predicting the pumping capability and viscous dissipation of two-dimensional flows in single-screw extrusion. The existing models are limited to dimensionless throughput rates of $\Pi_{V}\leq 2$. Especially for polymer melts with a distinct shear thinning nature (i.e., with a power law exponent in the range of 0.2 to 0.4), this means that the dimensionless pressure gradient is limited to approximately $\Pi_{p,z}\geq -0.5$. However, screw calculations of special screw designs, such as wave and energy-transfer screws, have shown heavily overridden screw zones, especially in the compression regions. This means that considerably large negative dimensionless pressure gradients (up to $\Pi_{p,z}\approx -0.9$) can be observed for power-law exponents of about 0.2 to 0.4. Since the existing models are not valid in this range, their predictions are insufficiently accurate for both throughput-pressure calculation and viscous dissipation. Further, the only general two-dimensional throughput-pressure model, presented by Pachner et al. [24], is discontinuous beyond its application range, featuring regions where it becomes infinite and abruptly changes the sign, and therefore has limited extrapolation capabilities. In summary, the existing models are not suitable for these special screw designs (wave and energy-transfer screws). Because wave and energy-transfer screws show interruptions of the screw flights and additionally in the sections around the wave peaks the screw channels are very shallow, two-dimensional modeling is preferred, which best represents the geometry of these screw channels.

**Figure 1:**
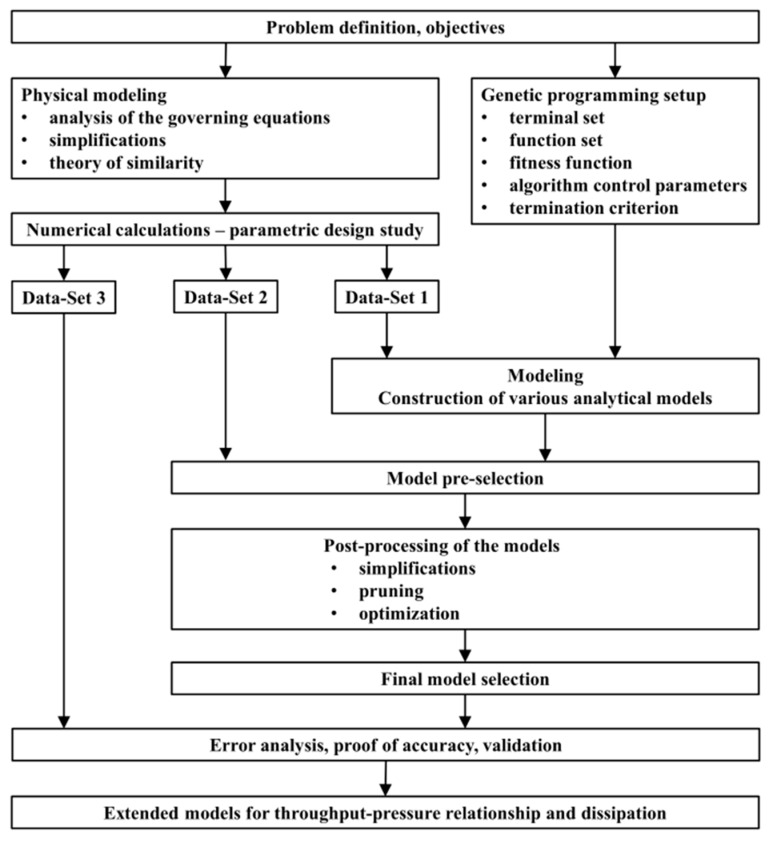
Schematic work flow chart of the extended analyses of throughput-pressure relationship and viscous dissipation.

In order to address these issues, we present general and continuous extended models that also consider negative dimensionless pressure gradients of up to $\Pi_{p,z}=-1.0$ for power-law exponents ranging from n=0.2 to n=1.0. Further, the proposed symbolic regression models cover an extended range for the screw-pitch ratio ($t/D_{b}=0.6$ to $t/D_{b}=2.4$). These models are suitable for both general-purpose screws and special screw designs, such as wave and energy-transfer screws. Additionally, they cover pressure-generating and pressure-reducing melt-conveying zones. The dissipation models can be used to calculate the axial melt-temperature profile and consequently the non-isothermal throughput-pressure behavior. First, a fundamental analysis of the governing equations was conducted based on the theory of similarity and then a comprehensive numerical parametric design study was carried out for the extended application range. Based on these results, approximation equations were derived by means of symbolic regression using genetic programming. We carried out comprehensive modeling and model pre-selection, analyzing the accuracy of the models on training data and on additional test data. These models were further simplified and optimized, and subsequently the final models were selected. Finally, we performed an error analysis, additionally validating the model with a third, independent validation set. Figure 1 shows a schematic of the work flow.

## 2. Flow Analysis

### 2.1. Geometric Modeling Approach

Modeling the melt-conveying zone of a single-screw extruder requires a geometric representation of the screw channel. In this work we used the following approach:

- The flat-plate assumption is applied for modeling the melt-conveying zone of a single-screw extruder. To this end, the helical screw channel is unwound and considered as a flat rectangular channel. The barrel surface is represented by an infinite flat plate. This approach, which ignores the influence of the curvature, is widely accepted in science and industry. Note that the channel curvature influences the polymer melt flow, as addressed by Sun and Rauwendaal [46] for Newtonian fluids, and by Roland et al. [47] for non-Newtonian fluids. The difference between flat-plate and cylindrical systems increases with increasing $h/D_{b}$ ratio. The flat-plate model with moving barrel is applicable for channel depth-to-diameter ratios $h/D_{b}<0.1$.
- The kinematics are reversed. This means that the screw is considered to be stationary and the barrel to be rotating with circumferential velocity $v_{b}$, calculated according to Equation (1) with the barrel diameter $D_{b}$ and the screw speed N. The circumferential speed is divided into a down-channel velocity $v_{b,z}$ (Equation (2)) and a cross-channel velocity $v_{b,x}$ (Equation (3)) component depending on the screw pitch angel $\varphi_{b}$ (Equation (4)), with barrel diameter $D_{b}$ and screw pitch t.
- The leakage flow over the flight gap δ is ignored. Usually, both the flight clearance and the flow through the clearance are small [8].
- The effect of the flights on the polymer melt flow is omitted. This approach is valid for shallow screw channels with h/w<0.1 [8]. Ignoring the effect of the flight flanks means that a two-dimensional velocity profile is considered with velocity components in the cross- and down-channel directions only.

**Figure 2:**
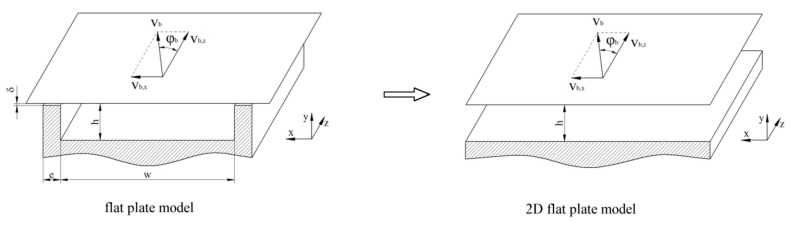
Flat-plate model of the unwound screw channel, (**left**) the rectangular flow channel; (**right**) the two-dimensional flat-plate model.

Finally, this leads to the two-dimensional flat-plate model with moving barrel, as shown in Figure 2.

(1) $$v_{b}=D_{b}\pi N.$$

(2) $$v_{b,z}=v_{b}\cos\left(\varphi_{b}\right),$$

(3) $$v_{b,x}=v_{b}\sin\left(\varphi_{b}\right),$$

with

(4) $$\varphi_{b}=arctan\left(\frac{t}{D_{b}\pi }\right).$$

### 2.2. Governing Equations

Determining the flow rate and viscous dissipation in the melt-conveying zone requires the balance equations to be solved by considering the boundary conditions. Detailed information on the deviation of the governing equations is provided in [40].

In addition to the geometric modeling approach, the following assumptions are made to solve the balance equations for the flow rate and viscous dissipation:

1. The fluid is incompressible;
2. The flow is stationary, fully developed, and isothermal;
3. Gravitational forces are ignored; and
4. The fluid is wall adhering.

Due to these simplifications, the velocity vector is reduced to Equation (5) with the down- and cross-channel velocities being functions of the channel height coordinate y only.

(5) $$v=\left(\begin{array}{c} v_{x}\left(y\right)\\ 0\\ v_{z}\left(y\right) \end{array}\right).$$

With these simplifications, the momentum equations are reduced to Equations (6) and (7) for the down- and cross-channel directions, respectively. Considering isothermal conditions uncouples the momentum equations from the energy equation. The latter is, therefore, no longer required for solving the velocity field. The viscous dissipation term can subsequently be evaluated based on the velocity profile derived by the momentum equations.

(6) $$\frac{\partial p}{\partial x}=\frac{\partial \tau_{yx}}{\partial y}.$$

(7) $$\frac{\partial p}{\partial z}=\frac{\partial \tau_{yz}}{\partial y}.$$

In order to be able to solve the simplified momentum equations for the down- and cross-channel velocity profiles, given a down-channel pressure gradient dp/dz, the boundary conditions must be defined. These are given by Equations (8)–(11) for a wall-adhering fluid, which is also well known as the no-slip condition. Additionally, it must be taken into account that the net cross-channel volumetric flow rate must be zero (Equation (12)), which determines the cross-channel pressure gradient. This means that the integral of the cross-channel velocity profile over the channel height must be zero.

(8) $$v_{x}\left(y=0\right)=0.$$

(9) $$v_{x}\left(y=h\right)=v_{b,x}.$$

(10) $$v_{z}\left(y=0\right)=0.$$

(11) $$v_{z}\left(y=h\right)=v_{b,z}.$$

(12) $${\dot{V}}_{x}=\int_{0}^{h}v_{x}\left(y\right)\cdot dy=0.$$

The simplified momentum equations (Equations (6) and (7)) are two non-linear partial differential equations coupled via the shear-rated dependent viscosity η. The shear-stress components are defined by the constitutive equation for the stress tensor τ (Equation (13)), which is the product of viscosity η and rate-of-deformation tensor D (Equation (14)). The latter is the symmetric part of the velocity gradient tensor L (Equation (15)).

(13) τ=2ηD.

(14) $$D=\frac{1}{2}\left(L+L^{T}\right).$$

(15) L=∇v.

For most polymer melts, the viscosity decreases with increasing shear rate. This behavior is called pseudo-plastic or shear-thinning. We consider this pseudo-plastic, non-Newtonian fluid behavior by using the power-law model (Equation (16)) with the consistency K, the power-law exponent n, and the magnitude of the shear-rate $\left|\dot{\gamma }\right|$. Using the power-law model, an error might be introduced because many polymer melts have a Newtonian plateau in the very low shear region. However, the error introduced by the very low shear-rate is not significant. This is because the high shear-rate regions determine flow rate and viscous dissipation [12]. Nevertheless, it is recommended to compute the power-law parameters for a representative shear-rate that appears in the screw channel to obtain best results. The magnitude of the shear-rate—a scalar quantity—is related to the second invariant of the rate-of-deformation tensor D and can be calculated according to Equation (17).

(16) $$\eta =K{\left|\dot{\gamma }\right|}^{n-1}.$$

(17) $$\left|\dot{\gamma }\right|=\sqrt{2\left(D:D\right)}.$$

Given the modeling assumptions, the power-law viscosity is reduced to Equation (18). Further, by applying the power-law model, the shear-stress components $\tau_{yx}$ and $\tau_{yz}$ are reduced to Equations (19) and (20), respectively.

(18) $$\eta =K{\left[{\left(\frac{\partial v_{x}}{\partial y}\right)}^{2}+{\left(\frac{\partial v_{z}}{\partial y}\right)}^{2}\right]}^{\frac{n-1}{2}}.$$

(19) $$\tau_{yx}=K{\left[{\left(\frac{\partial v_{x}}{\partial y}\right)}^{2}+{\left(\frac{\partial v_{z}}{\partial y}\right)}^{2}\right]}^{\frac{n-1}{2}}\frac{\partial v_{x}}{\partial y}.$$

(20) $$\tau_{yz}=K{\left[{\left(\frac{\partial v_{x}}{\partial y}\right)}^{2}+{\left(\frac{\partial v_{z}}{\partial y}\right)}^{2}\right]}^{\frac{n-1}{2}}\frac{\partial v_{z}}{\partial y}.$$

Now we take the shear-stress components (Equations (19) and (20)) and apply them to the simplified momentum equations (Equations (6) and (7)), which leads us to our final momentum equations for the cross-channel direction (Equation (21)) and the down-channel direction (Equation (22)).

(21) $$\frac{\partial p}{\partial x}=\frac{\partial }{\partial y}\left(\eta \left(y\right)\frac{\partial v_{x}}{\partial y}\right)=\frac{\partial }{\partial y}\left\{K{\left[{\left(\frac{\partial v_{x}}{\partial y}\right)}^{2}+{\left(\frac{\partial v_{z}}{\partial y}\right)}^{2}\right]}^{\frac{n-1}{2}}\frac{\partial v_{x}}{\partial y}\right\}.$$

(22) $$\frac{\partial p}{\partial z}=\frac{\partial }{\partial y}\left(\eta \left(y\right)\frac{\partial v_{z}}{\partial y}\right)=\frac{\partial }{\partial y}\left\{K{\left[{\left(\frac{\partial v_{x}}{\partial y}\right)}^{2}+{\left(\frac{\partial v_{z}}{\partial y}\right)}^{2}\right]}^{\frac{n-1}{2}}\frac{\partial v_{z}}{\partial y}\right\}.$$

Using these momentum equations, the boundary conditions, and the supplementary constraint of zero net cross-channel flow, we can determine the down- and cross-channel velocities by means of numerical methods as functions of the channel-height coordinate y and the cross-channel pressure gradient ∂p/∂x. The flow rate and the viscous dissipation can be evaluated based on the velocity profile. The volumetric flow rate is the integral of the down-channel velocity profile $v_{z}\left(y\right)$ over the cross-sectional area, as given by Equation (23), with i as the number of parallel screw channels and w as the channel width. The specific viscous dissipation per unit volume is given by ${\dot{q}}_{diss}=\tau :L$. The simplifications above reduce the specific dissipation to Equation (24). The total dissipation over the cross-channel area is the integral of the specific dissipation over the cross-channel area (Equation (25)).

(23) $$\dot{V}=iw\int_{0}^{h}v_{z}\left(y\right)dy.$$

(24) $${\dot{q}}_{diss}=\tau_{yx}\frac{\partial v_{x}}{\partial y}+\tau_{yz}\frac{\partial v_{z}}{\partial y}=\eta \left[{\left(\frac{\partial v_{x}}{\partial y}\right)}^{2}+{\left(\frac{\partial v_{z}}{\partial y}\right)}^{2}\right].$$

(25) $${\dot{Q}}_{Diss}=iw\int_{0}^{h}{\dot{q}}_{diss}\left(y\right)dy.$$

### 2.3. Theory of Similarity

#### 2.3.1. Transformation of Governing Equations into Dimensionless Form

In the next step, the governing equations are transformed into dimensionless form by applying the theory of similarity and dimensional analysis. In the fields of fluid dynamics and heat engineering, the theory of similarity is a common tool for transforming phenomena observed at laboratory scale to the scale of real-world applications [48]. In this work, we sought to obtain generalized results that can be applied to any arbitrary real-world screw design by transforming the governing equations into dimensionless form. In fact, our solutions are dimensionless—that is, indirect. Generally, two systems that are described by the same dimensionless quantities are similar. This means that they are described by the same physics, but can operate under different operating conditions. Hence, the dimensionless solution for a specific set of independent dimensionless influencing parameters applies to all dimensional variations that result in this particular set of dimensionless parameters. Moreover, by applying the theory of similarity, the number of independent influencing parameters can be reduced dramatically and the scales of the independent and dependent variables are harmonized.

Scaling partial differential equations (i.e., transforming them into dimensionless form) requires the following steps [49]:

1. Identify the independent and dependent variables.
2. Introduce characteristic values of the independent and dependent variables and make them dimensionless.
3. Insert the dimensionless variables into the governing equations and derive a model that has dimensionless variables only.
4. Make each term dimensionless. Divide by the coefficient in front of any term (preferably by the coefficient in front of the term with the highest derivative).
5. When not yet fixed, define a characteristic quantity for the characteristic values introduced in step (2).

For the problem under consideration, the down- and cross-channel velocities ($v_{z}$ and $v_{x}$) are the dependent variables and the channel height coordinate y is the independent variable. Hence, we introduce the dimensionless down-channel velocity $\nu_{z}$ (Equation (26)), the dimensionless cross-channel velocity $\nu_{x}$ (Equation (27)), and the dimensionless channel-height coordinate ξ (Equation (28)). The down-channel velocity at the top plate (barrel surface) $v_{b,z}$ is chosen as the characteristic velocity, and the channel height h as the characteristic length.

(26) $$\nu_{z}=\frac{v_{z}}{v_{b,z}}.$$

(27) $$\nu_{x}=\frac{v_{x}}{v_{b,z}}.$$

(28) $$\xi =\frac{y}{h}.$$

In the next step, these dimensionless variables are introduced into our governing equations: First, the dimensionless variables are applied to the shear-rate-dependent viscosity η (see Equation (18)), which results in Equation (29) for the dimensionless viscosity.

(29) $$\eta^{*}=\frac{\eta h^{n-1}}{Kv_{b,z}^{n-1}}={\left|{\dot{\gamma }}^{*}\right|}^{n-1}={\left[{\left(\frac{\partial \nu_{z}}{\partial \xi }\right)}^{2}+{\left(\frac{\partial \nu_{x}}{\partial \xi }\right)}^{2}\right]}^{\frac{n-1}{2}}.$$

Further, the dimensionless variables are applied to the momentum equations to obtain the dimensionless momentum equations in the down-channel (Equation (30)) and cross-channel (Equation (31)) direction. Making the remaining terms dimensionless yields the dimensionless down-channel pressure gradient $\Pi_{p,z}$ (Equation (32)) and the dimensionless cross-channel pressure gradient $\Pi_{p,x}$ (Equation (33)).

(30) $$6\Pi_{p,z}=\frac{\partial }{\partial \xi }\left(\eta^{*}\frac{\partial \nu_{z}}{\partial \xi }\right)=\frac{\partial }{\partial \xi }\left\{{\left[{\left(\frac{\partial \nu_{z}}{\partial \xi }\right)}^{2}+{\left(\frac{\partial \nu_{x}}{\partial \xi }\right)}^{2}\right]}^{\frac{n-1}{2}}\frac{\partial \nu_{z}}{\partial \xi }\right\}.$$

(31) $$6\Pi_{p,x}=\frac{\partial }{\partial \xi }\left(\eta^{*}\frac{\partial \nu_{x}}{\partial \xi }\right)=\frac{\partial }{\partial \xi }\left\{{\left[{\left(\frac{\partial \nu_{z}}{\partial \xi }\right)}^{2}+{\left(\frac{\partial \nu_{x}}{\partial \xi }\right)}^{2}\right]}^{\frac{n-1}{2}}\frac{\partial \nu_{x}}{\partial \xi }\right\}.$$

(32) $$\Pi_{p,z}=\frac{\frac{\partial p}{\partial z}h^{n+1}}{6Kv_{b,z}^{n}}.$$

(33) $$\Pi_{p,x}=\frac{\frac{\partial p}{\partial x}h^{n+1}}{6Kv_{b,z}^{n}}.$$

Similarly, the boundary conditions are made dimensionless, which results in Equations (34) to (37). The constraint that the net cross-channel flow must be zero is also transformed into dimensionless form (Equation (38)).

(34) $$\nu_{x}\left(\xi =0\right)=0.$$

(35) $$\nu_{x}\left(\xi =1\right)=\frac{v_{b,x}}{v_{b,z}}=\tan\left(\varphi_{b}\right)=\frac{t}{D_{b}\pi }.$$

(36) $$\nu_{z}\left(\xi =0\right)=0.$$

(37) $$\nu_{z}\left(\xi =1\right)=1.$$

(38) $$\Pi_{V,x}=\int_{0}^{1}\nu_{x}\left(\xi \right)\cdot d\xi =0.$$

Using the dimensionless equations of motions and numerical methods allows the dimensionless down- and cross-channel velocity profiles to be determined for a given down-channel pressure gradient. Additionally, the zero net cross-channel flow constraint determines the dimensionless cross-channel pressure gradient. Nevertheless, there is an effect of the down-channel flow due to the shear-rate-dependent viscosity. Since these results allow the flow rate and viscous dissipation to be determined, the flow rate and viscous dissipation are also transformed into dimensionless form. The dimensionless flow rate is defined by Equation (39) and can be evaluated according to Equation (40) based on the dimensionless down-channel velocity profile. The dimensionless specific dissipation is defined and calculated according to Equation (41). In analogy to the flow rate, the dimensionless total dissipation is defined by Equation (42) and can be evaluated according to Equation (43).

(39) $$\Pi_{V}=\frac{2\dot{V}}{iwhv_{b,z}}.$$

(40) $$\Pi_{V}=2\int_{0}^{1}\nu_{z}\left(\xi \right)d\xi .$$

(41) $$\pi_{q}\left(\xi \right)=\frac{{\dot{q}}_{diss}h^{n+1}}{Kv_{b,z}^{n+1}}=\eta^{*}\left[{\left(\frac{\partial \nu_{z}}{\partial \xi }\right)}^{2}+{\left(\frac{\partial \nu_{x}}{\partial \xi }\right)}^{2}\right].$$

(42) $$\Pi_{Q}=\frac{{\dot{Q}}_{Diss}h^{n}}{iwKv_{b,z}^{n+1}}.$$

(43) $$\Pi_{Q}=\int_{0}^{1}\pi_{q}\left(\xi \right)d\xi =\int_{0}^{1}{\left[{\left(\frac{\partial \nu_{z}}{\partial \xi }\right)}^{2}+{\left(\frac{\partial \nu_{x}}{\partial \xi }\right)}^{2}\right]}^{\frac{n+1}{2}}d\xi .$$

In summary, by applying the theory of similarity, the governing equations are transformed into dimensionless form, which results in three independent dimensionless influencing parameters:

1. The dimensionless pressure gradient $\Pi_{p,z}$;
2. The power-law exponent n; and
3. The screw-pitch ratio $t/D_{b}$.

#### 2.3.2. Set-Up of Parametric Design Study

Taking the findings of the dimensional analysis into account, we created three different sets of independent design points by varying the dimensionless input parameters $\Pi_{p,z}$, n, and $t/D_{b}$. The first data set was used as training set for symbolic regression, and the second as test set. The first and second sets together were used for model selection and post-processing, and the third was used as a validation set to obtain a more objective and unbiased evaluation of the final model. The symbolic regression is described in detail in the Section Analytical Approximation. For Data Set 1 and Data Set 2, the ranges of the screw-pitch ratio $t/D_{b}$, the power-law exponent n, and the dimensionless down-channel pressure gradient $\Pi_{p,z}$ are given in Table 1 and Table 2, respectively. Note that Data Set 2 is related to a data set used in our previous study [40]. For the present study, the range of the screw-pitch ratio was extended to $t/D_{b,max}=2.4$. Further, we extended the range of the dimensionless down-channel pressure gradient. In previous studies [24,39,40], the lower limit for the dimensionless down-channel pressure gradient was chosen to achieve dimensionless throughputs of approximately $\Pi_{V,max}\geq 2.0$. This would mean that the throughput is approximately twice the pure drag flow. For polymer melts with distinct shear-thinning nature, it follows that the dimensionless down-channel pressure gradient is limited to approximately $\Pi_{p,z}\geq -0.5$. Screw calculations have shown that this limit is insufficient for special screw designs, such as wave- and energy-transfer screws. Hence, for this work the lower limit of the dimensionless down-channel pressure gradient of Data Set 1 was extended and fixed to $\Pi_{p,z,min}=-1.0$ for all combinations of the screw pitch ratio $t/D_{b}$ and power-law exponents n. The resulting range of $\Pi_{p,z}$ was divided into 40 equidistant divisions. For Data Set 2, the limitation remained the same, and the resulting range of $\Pi_{p,z}$ was divided into 60 equidistant divisions. For Data Set 3, an independent validation set was generated. Within the parameter range of Data Set 1, nine and six random values were chosen, respectively, for the screw-pitch ratio $t/D_{b}$ and the power-law exponent n (see Table 3). For each combination of these two parameters, the resulting range of dimensionless down-channel pressure gradients was divided into 17 equidistant divisions. Table 4 summarizes the number of variations for each dimensionless influencing parameter and the resulting total number of independent design points for each data set. The chosen range of the dimensionless influencing parameters covers almost all materials, operating conditions, and screw geometries possible in polymer extrusion.

## 3. Numerical Calculations

Applying the theory of similarity reduced the number of influencing parameters dramatically to three: (i) $\Pi_{p,z}$, (ii) n, and (iii) $t/D_{b}$. These describe completely the physics of the underlying problem. Determining the dimensionless target values—the dimensionless throughput $\Pi_{V}$ and dimensionless dissipation $\Pi_{Q}$—first requires the dependent dimensionless variables to be calculated. These are the dimensionless down-channel and cross-channel velocities. To this end, the dimensionless momentum equations in the down-channel and cross-channel directions (Equations (30) and (31)) must be solved. Due to the shear-rate-dependent viscosity, these are two non-linear coupled partial differential equations with unknown analytical solution that must be solved by numerical methods. Partial differential equations can be solved by various numerical methods, for instance, the finite difference method (FDM), the finite element method (FEM), and the shooting method. It has been shown that all three methods are suitable for solving this problem [39]. For this work, we chose the shooting method to compute the numerical solutions.

### 3.1. Numerical Solution

The basic idea of the shooting method is to transform the boundary value problem into an initial value problem. To this end, the momentum equations are transformed into explicit form for the velocity gradients. First, both dimensionless momentum equations (Equations (30) and (31)) are integrated over the dimensionless channel height, which yields Equations (44) and (45).

(44) $$6\Pi_{p,z}\xi +C_{1}=\eta^{*}\frac{\partial \nu_{z}}{\partial \xi }.$$

(45) $$6\Pi_{p,x}\xi +C_{2}=\eta^{*}\frac{\partial \nu_{x}}{\partial \xi }.$$

Next, the shear-rate-dependent viscosity is substituted. For this, the two integrated momentum equations are first squared and then added to obtain Equation (46). This equation can be re-formulated by use of Equation (29) to express the dimensionless viscosity $\eta^{*}$ in the form of Equation (47).

(46) $$\eta^{*2}\left[{\left(\frac{\partial \nu_{z}}{\partial \xi }\right)}^{2}+{\left(\frac{\partial \nu_{x}}{\partial \xi }\right)}^{2}\right]={\left(6\Pi_{p,z}\xi +C_{1}\right)}^{2}+{\left(6\Pi_{p,x}\xi +C_{2}\right)}^{2}.$$

(47) $$\eta^{*}={\left[{\left(6\Pi_{p,z}\xi +C_{1}\right)}^{2}+{\left(6\Pi_{p,x}\xi +C_{2}\right)}^{2}\right]}^{\frac{n-1}{2n}}.$$

This expression for the dimensionless viscosity $\eta^{*}$ is used in Equations (44) and (45). By further rearrangement, the momentum equations are transformed into explicit form for the down-channel and cross-channel velocity gradients:

(48) $$\frac{\partial \nu_{z}}{\partial \xi }=\frac{1}{\eta^{*}}\left(6\Pi_{p,z}\xi +C_{1}\right).$$

(49) $$\frac{\partial \nu_{x}}{\partial \xi }=\frac{1}{\eta^{*}}\left(6\Pi_{p,x}\xi +C_{2}\right).$$

For a given set of dimensionless input parameters ($\Pi_{p,z}$, n, and $t/D_{b}$), the integration constants $C_{1}$ and $C_{2}$ and the dimensionless cross-channel pressure gradient $\Pi_{p,x}$, are unknown and must be determined. Physically, this means that the integration constants $C_{1}$ and $C_{2}$ are the dimensionless wall shear stresses at the screw surface in the down-channel and cross-channel directions, respectively. Determining the unknowns requires initial estimates for $C_{1}$, $C_{2}$, and $\Pi_{p,x}$, which are taken from the Newtonian case given by Equations (50) to (52).

(50) $$C_{1}=1-3\Pi_{p,z}.$$

(51) $$C_{2}=-2\tan\left(\varphi_{b}\right)=-2\frac{t}{D_{b}\pi }.$$

(52) $$\Pi_{p,x}=\tan\left(\varphi_{b}\right)=\frac{t}{D_{b}\pi }.$$

With these initial values, the dimensionless velocity profiles can be computed by integrating the velocity gradients over the dimensionless channel height (Equations (53) and (54)). Note that the initial values are the boundary conditions at the screw wall.

(53) $$\nu_{z}\left(\xi \right)=\nu_{z}\left(\xi =0\right)+\int_{0}^{1}\frac{\partial \nu_{z}}{\partial \xi }d\xi .$$

(54) $$\nu_{x}\left(\xi \right)=\nu_{x}\left(\xi =0\right)+\int_{0}^{1}\frac{\partial \nu_{x}}{\partial \xi }d\xi .$$

The integration is done numerically using the Simpson rule. Unless the initial values are perfect, the boundary conditions at the top (barrel) wall and the condition that the net cross-channel flow must be zero will not be met, and hence there will be some residuals. We applied a Newton-Raphson scheme (Equation (55)) to iteratively solve the unknowns until the solution has converged.

(55) $$x_{n+1}=x_{n}-J{\left(x_{n}\right)}^{-1}\left[f\left(x_{n}\right)-f\left(x\right)\right].$$

For this problem, the vector of the unknown variables x contains the integration constants $C_{1}$ and $C_{2}$ and the dimensionless cross-channel pressure gradient $\Pi_{p,x}$ (Equation (56)). The function vector f contains both dimensionless boundary conditions at the top wall and the dimensionless cross-channel volume flow rate (Equation (57)). $J\left(x_{n}\right)$ is the Jacobian containing all partial derivatives of the three functions in f with respect to the three variables in x (Equation (58)). The partial derivatives are determined numerically by slightly varying $C_{1}$, $C_{2}$, and $\Pi_{p,x}$ and re-computing the velocity profiles and the cross-channel volume flow rate. Finally, the new values of the unknown variables are calculated according to Equation (59) for the next iteration. This procedure converges rapidly and requires between five to ten iterations, depending on the dimensionless influencing parameters. With the converged solution for the velocity profiles, the dimensionless volume flow rate $\Pi_{V}$ (Equation (40)) and the dimensionless dissipation $\Pi_{Q}$ (Equation (43)) are evaluated using the Simpson rule for all numerical integrations.

(56) $$x=\left(\begin{array}{c} C_{1}\\ C_{2}\\ \Pi_{p,x} \end{array}\right).$$

(57) $$f=\left(\begin{array}{c} \nu_{z}\left(\xi =1\right)\\ \nu_{x}\left(\xi =1\right)\\ \Pi_{V,x} \end{array}\right)=\left(\begin{array}{c} \nu_{z,1}\\ \nu_{x,1}\\ \Pi_{V,x} \end{array}\right).$$

(58) $$J\left(x\right)=\left[\begin{array}{ccc} \frac{\partial \nu_{z,1}}{\partial C_{1}} & \frac{\partial \nu_{z,1}}{\partial C_{2}} & \frac{\partial \nu_{z,1}}{\partial \Pi_{p,x}}\\ \frac{\partial \nu_{x,1}}{\partial C_{1}} & \frac{\partial \nu_{x,1}}{\partial C_{2}} & \frac{\partial \nu_{x,1}}{\partial \Pi_{p,x}}\\ \frac{\partial \Pi_{V,x}}{\partial C_{1}} & \frac{\partial \Pi_{V,x}}{\partial C_{2}} & \frac{\partial \Pi_{V,x}}{\partial \Pi_{p,x}} \end{array}\right].$$

(59) $$\left(\begin{array}{c} C_{1,n+1}\\ C_{2,n+1}\\ \Pi_{p,x,n+1} \end{array}\right)=\left(\begin{array}{c} C_{1,n}\\ C_{2,n}\\ \Pi_{p,x,n} \end{array}\right)-J{\left(x_{n}\right)}^{-1}\left[\left(\begin{array}{c} \nu_{z,1,n}\\ \nu_{x,1,n}\\ \Pi_{V,x,n} \end{array}\right)-\left(\begin{array}{c} 1\\ t/\left(D_{b}\pi \right)\\ 0 \end{array}\right)\right].$$

The parameter setup chosen for the numerical simulations is given in Table 5. The dimensionless channel height was divided into 1000 equidistant segments to obtain a total of 1001 nodes. As abortion criterion, the dimensionless volume flow rate was chosen. The solution was considered converged if the change in the dimensionless volume flow rate ${\Delta \Pi }_{V}$ between two iterations was $<10^{-8}$. Both the number of nodes and the abortion criterion were proven to be sufficient to reach a mesh-independent solution for throughput and dissipation. Furthermore, for numerical reasons, the dimensionless viscosity was limited to values between a specified maximum and minimum. These limits were also tested and had no influence on $\Pi_{V}$ and $\Pi_{Q}$, even for low power-law exponents n and high dimensionless pressure gradients $\Pi_{p,z}$.

### 3.2. Results of the Numerical Calculations

**Figure 3:**
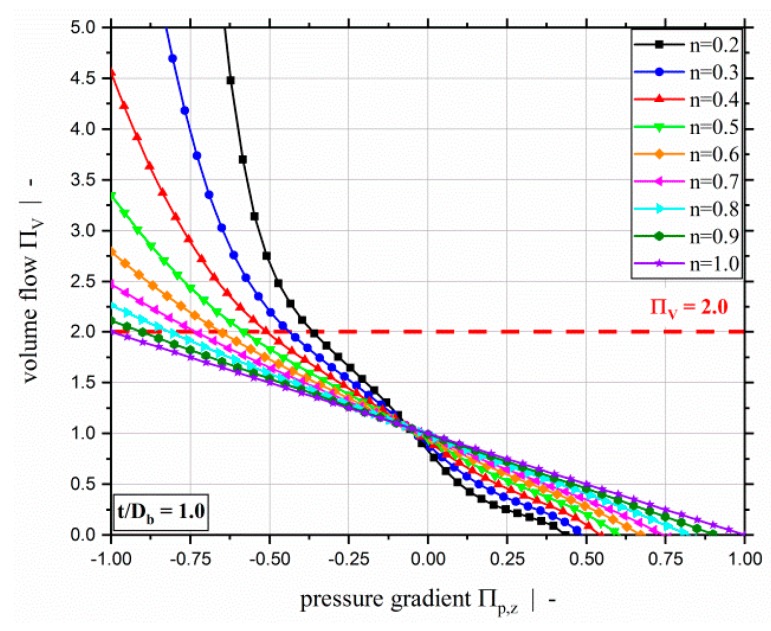
Dimensionless volume flow $\Pi_{V}$ over dimensionless down-channel pressure gradient $\Pi_{p,z}$ of a two-dimensional flow for a screw-pitch ratio of $t/D_{b}=1.0$ and various power-law exponents. The dashed red line highlights the limit of previous models at $\Pi_{V}=2$.

**Figure 4:**
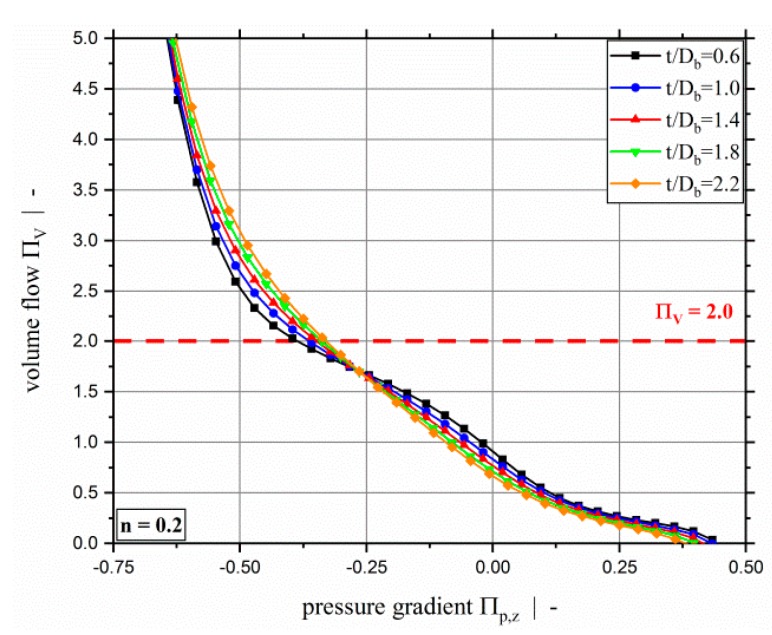
Dimensionless volume flow $\Pi_{V}$ over dimensionless down-channel pressure gradient $\Pi_{p,z}$ of a two-dimensional flow for a power-law exponent of n=0.2 and various screw-pitch ratios. Results are shown up to a dimensionless flow rate of $\Pi_{V}=5$. The dashed red line highlights the limit of previous models at $\Pi_{V}=2$.

**Figure 5:**
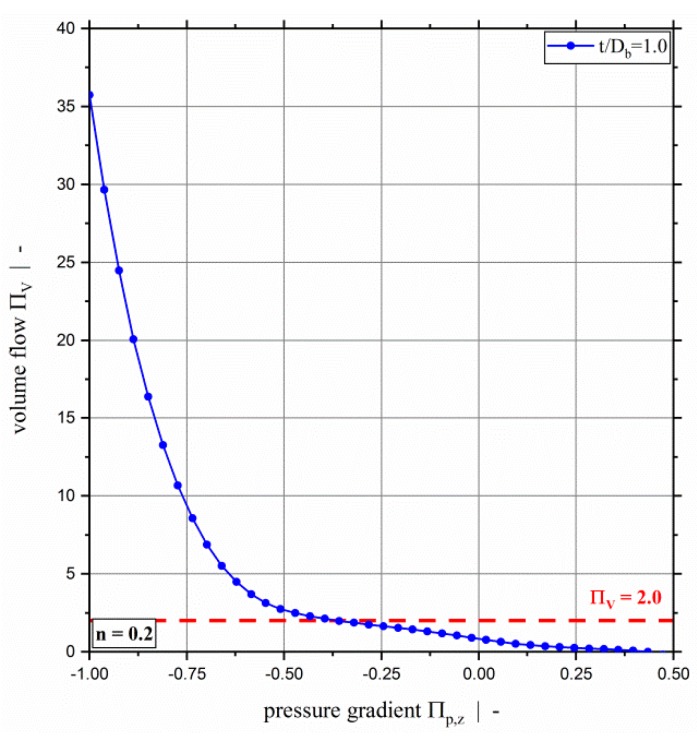
Dimensionless volume flow $\Pi_{V}$ over dimensionless down-channel pressure gradient $\Pi_{p,z}$ of a two-dimensional flow for a power-law exponent of n=0.2 and a screw-pitch ratio of $t/D_{b}=1.0$. The dashed red line highlights the limit of previous models at $\Pi_{V}=2$.

The comprehensive parametric design study yielded numerical solutions for the dimensionless volume flow rate $\Pi_{V}$ and dissipation $\Pi_{Q}$ as functions of the dimensionless independent influencing parameters $\Pi_{p,z}$, n, and $t/D_{b}$. Additionally, it provided results for the dimensionless cross-channel pressure gradient $\Pi_{p,x}$. Figure 3 plots the dimensionless volume flow rate over the dimensionless down-channel pressure gradient for a screw-pitch ratio of $t/D_{b}=1.0$ and various power-law exponents n. It can be clearly seen that the throughput-pressure relationship becomes increasingly non-linear and pressure-sensitive with decreasing power-law exponent. Previous research [8,19,22,24] provided results for $\Pi_{V}\leq 2.0$ (highlighted by a dashed red line). Our results for this area show very good agreement with the previous two-dimensional analyses. Here, we also present results for dimensionless pressure gradients $\Pi_{p,z}\geq -1.0$ for over ridden zones. For polymer melts with distinct shear-thinning nature, this can give rise to dimensionless volume flow rates that are significantly higher than $\Pi_{V}\leq 2.0.$ Figure 4 shows the dimensionless throughput-pressure relationship for various screw-pitch ratios and a power-law exponent of n=0.2, and highlights the significant increase in dimensionless volume flow rate for strongly over-ridden screw zones. Additionally, it can be seen that the dimensionless throughput decreases with increasing screw-pitch ratio for both pressure-generating and slightly over-ridden screw zones. For highly over-ridden screw zones, the dimensionless throughput increases with increasing screw-pitch ratio. This effect is due to the influence of the cross-channel flow on the shear-rate-dependent viscosity. For a distinctly negative dimensionless down-channel pressure gradient (exceeding $\Pi_{p,z}\approx -0.5$ for n=0.2), the effect of the screw-pitch ratio on the dimensionless volume flow rate decreases because the down-channel pressure flow dominates the flow behavior. In Figure 5, the dimensionless flow rate as a function of the dimensionless down-channel pressure gradient is given for a power-law exponent n=0.2 and a screw-pitch ratio $t/D_{b}=1.0$ for the whole range of the dimensionless pressure gradient. For a dimensionless down-channel pressure gradient of $\Pi_{p,z}=-1.0$, the dimensionless volume flow rate exceeds values of $\Pi_{V}=35$.

**Figure 6:**
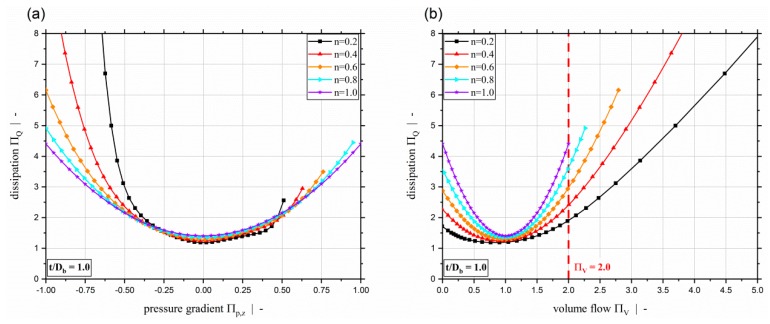
Dimensionless dissipation $\Pi_{Q}$ over dimensionless down-channel pressure gradient $\Pi_{p,z}$ (**a**) and over the dimensionless volume flow rate $\Pi_{V}$ (**b**) for various power-law exponents n and a screw-pitch ratio of $t/D_{b}=1.0$.

**Figure 7:**
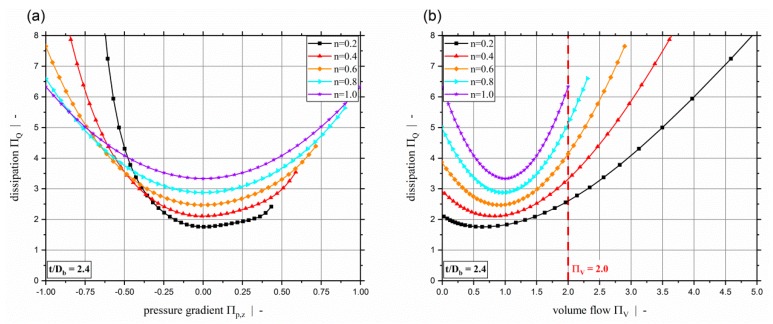
Dimensionless dissipation $\Pi_{Q}$ over dimensionless down-channel pressure gradient $\Pi_{p,z}$ (**a**) and over the dimensionless volume flow rate $\Pi_{V}$ (**b**) for various power-law exponents n and a screw-pitch ratio of $t/D_{b}=2.4$.

**Figure 8:**
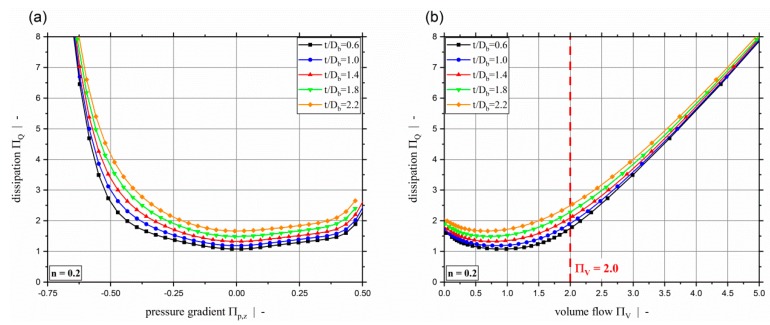
Dimensionless dissipation $\Pi_{Q}$ over dimensionless down-channel pressure gradient $\Pi_{p,z}$ (**a**) and over the dimensionless volume flow rate $\Pi_{V}$ (**b**) for various screw-pitch ratios $t/D_{b}$.

**Figure 9:**
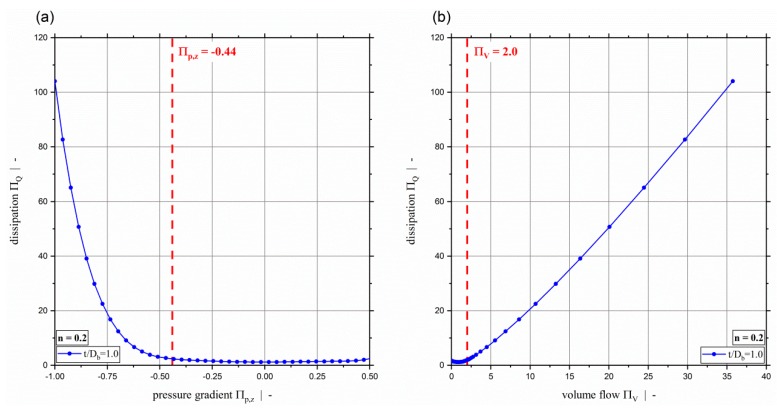
Dimensionless dissipation $\Pi_{Q}$ over dimensionless down-channel pressure gradient $\Pi_{p,z}$ (**a**) and over the dimensionless volume flow rate $\Pi_{V}$ (**b**) for a power-law exponent of n=0.2 and a screw-pitch ratio of $t/D_{b}=1.0$. For the parameter setup shown, the dashed red lines indicate (**a**) the limit for the dimensionless down-channel pressure gradient of the previous study and (**b**) the limit for the dimensionless volume flow rate.

Figure 6 plots the dimensionless dissipation $\Pi_{Q}$ for various power-law exponents and a screw-pitch ratio of $t/D_{b}=1.0$. Due to the relation between the dimensionless throughput and the dimensionless down-channel pressure gradient, the dimensionless dissipation can be given both relative to the dimensionless down-channel pressure gradient (see Figure 6a) and relative to the dimensionless volume flow rate (Figure 6b). Generally, it can be seen that the dimensionless dissipation reaches its minimum for pure drag flow ($\Pi_{p,z}=0$). For moderate dimensionless pressure gradients (positive and negative), the dissipation decreases with decreasing power-law exponent. For higher magnitudes, the dissipation increases with decreasing power-law exponents. Especially for $\Pi_{p,z}\leq -0.5$, the dissipation becomes highly dependent on the dimensionless down-channel pressure gradient and the power-law exponent. Considering constant dimensionless volume flow rates, the dissipation decreases with decreasing power-law exponent. Figure 6b indicates the limitation of $\Pi_{V}\leq 2.0$ of our previous study [40]. Figure 7 shows the dimensionless dissipation for various power-law exponents n and $t/D_{b}=2.4$. Comparison of the results for $t/D_{b}=2.4$ with those for $t/D_{b}=1.0$ shows that the influence of the power-law exponent on the dissipation increases with increasing screw-pitch ratio. Especially for moderate dimensionless pressure gradients, this effect is significant. This is due to the effect of the cross-channel flow on the shear rate and thus on the viscosity. With increasing screw-pitch ratio the shear rate increases, which leads to an increased influence of the shear-thinning effect on the viscosity. Figure 8 plots the dimensionless dissipation for a power-law exponent n=0.2 and various screw-pitch ratios $t/D_{b}$. For both constant dimensionless down-channel pressure gradient and volume flow rate, dissipation increases with increasing screw-pitch ratio. This is due to an increased cross-channel flow that results in an increased shear-rate, and thus an increase in viscous dissipation. As already observed for the dimensionless volume flow rate, the effect of the screw-pitch ratio on the dissipation decreases for strongly over-ridden screw zones because the flow behavior is dominated by the pressure flow. Note that in our previous study [40] the dimensionless down-channel pressure gradient for n=0.2 and over-ridden screw zones was limited to the range $\Pi_{p,z,min}\approx \left[-0.55;-0.43\right]$ depending on the screw-pitch ratio. For n=0.2 and $t/D_{b}=1.0$, the dissipation for the whole range of $\Pi_{p,z}$ is given in Figure 9. For this combination, the dimensionless down-channel pressure gradient was limited to $\Pi_{p,z,min}=-0.44$. Below this limit the dissipation becomes strongly dependent on the dimensionless down-channel pressure gradient, exceeding a dimensionless dissipation of $\Pi_{Q}=100$ for $\Pi_{p,z}=-1.0$; in contrast, the maximum dimensionless dissipation is $\Pi_{Q,max}=5.75$ for the traditional limit.

For small power-law exponents n and large negative dimensionless down-channel pressure gradients $\Pi_{p,z}$, we observed that the influence of the screw-pitch ratio on the dimensionless throughput and dimensionless dissipation becomes minimal and vanishes. This indicates that the pressure flow dominates the total flow behavior. Ignoring the cross-channel flow and assuming that the down-channel pressure flow dominates the complete flow behavior allows the expression for the total dimensionless flow rate $\Pi_{V}$ to be transformed into Equation (60). This represents a drag-flow component and the dimensionless pressure flow component $\Pi_{V,p}$ (Equation (61)) for a one-dimensional flow of power-law fluids. The total dimensionless dissipation $\Pi_{Q}$ can be transformed into Equation (62), which represents the dimensionless dissipation of one-dimensional pure pressure flow.

(60) $$\Pi_{V}=1+\Pi_{V,p}.$$

(61) $$\Pi_{V,p}=-\mathrm{sign}\left(\Pi_{p,z}\right)\frac{3^{\frac{1}{n}}n}{2n+1}{\left|\Pi_{p,z}\right|}^{\frac{1}{n}}.$$

(62) $$\Pi_{Q}=\frac{3^{\frac{n+1}{n}}n}{2n+1}{\left|\Pi_{p,z}\right|}^{\frac{n+1}{n}}.$$

**Figure 10:**
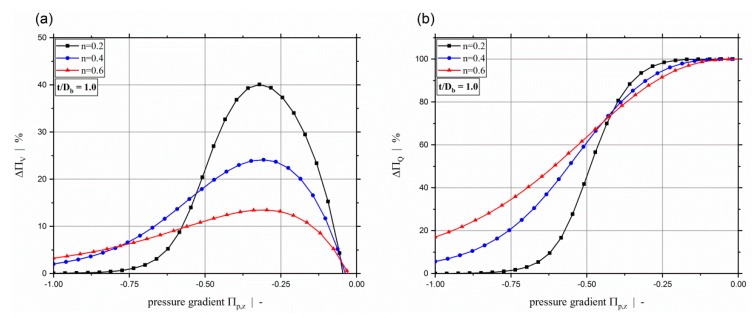
Difference between numerical data set and limiting approach for large negative dimensionless pressure gradients according to Equations (60) to (62), expressed in percentages for various power-law exponents: (**a**) dimensionless throughput $\Pi_{V}$, (**b**) dimensionless dissipation $\Pi_{Q}$.

Figure 10 illustrates the difference between the one-dimensional pressure-flow approach and the numerical solutions for negative dimensionless down-channel pressure gradients. It can clearly be seen that for n=0.2 the flow is dominated by the pressure flow for $\Pi_{p,z}\leq -0.75$. With higher power-law exponents, the shear-thinning effect decreases, and thus larger negative dimensionless down-channel pressure gradients are needed to reach the point where pure pressure flow dominates the flow behavior.

**Figure 11:**
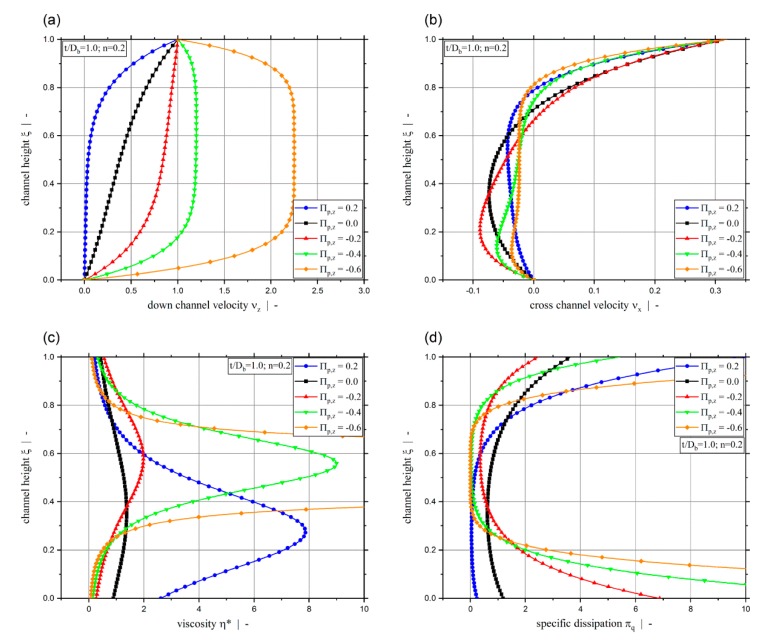
Dimensionless channel height ξ versus dimensionless down-channel velocity $\nu_{z}$ (**a**), dimensionless cross-channel velocity $\nu_{x}$ (**b**), dimensionless viscosity $\eta^{*}$ (**c**), and dimensionless specific dissipation $\pi_{q}$ (**d**) for various dimensionless down-channel pressure gradients $\Pi_{p,z}$, n=0.2, and $t/D_{b}=1.0$.

To gain a deeper understanding of the strong influence of the dimensionless pressure gradient on the throughput and dissipation for small power-law exponents, Figure 11 plots the down-channel velocity (a), cross-channel velocity (b), dimensionless viscosity (c), and dimensionless dissipation (d) over the dimensionless channel height for a power-law exponent n=0.2 and a screw-pitch ratio of $t/D_{b}=1.0$ for various pressure gradients. It can be seen that decreasing the dimensionless down-channel pressure gradient from $\Pi_{p,z}=-0.4$ to $\Pi_{p,z}=-0.6$ increases the down-channel velocity disproportionately. For $\Pi_{p,z}=-0.6$ in the center of the screw-channel, the flow in the down- and cross-channel directions resembles plug flow. The shear rate is therefore close to zero and the viscosity very high. On the other side, near the walls, the viscosity is close to zero and the shear rate very high. This can also be observed in the dimensionless dissipation profile, where dissipation increases near the walls and decreases in the center with increasing magnitude of the dimensionless pressure gradient. This effect can be attributed to the shear stress distribution of pure pressure flow. For pure pressure flow, it follows that the shear stress is linear over the channel height, being maximum at the walls and zero in the center. According to the power-law model, the shear-rate is related to the shear-stress by $\left|\dot{\gamma }\right|\sim \tau^{1/n}$. This means that for low power-law exponents, high shear-rates (velocity gradients) are expected near the wall and low shear-rates near the center. However, the down-channel flow in the metering section is affected by the cross-channel flow. However, with increasing dimensionless down-channel pressure gradient, this influence decreases and the down-channel flow dominates the overall behavior. Following, this almost causes plug flow in the center, not only for the down-channel, but also for the cross-channel flow, due to the coupling via the shear-rate dependent viscosity.

**Figure 12:**
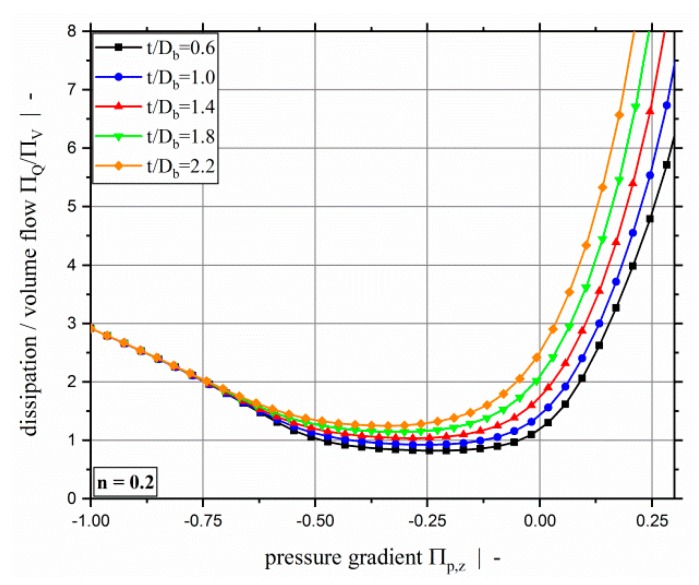
Dimensionless dissipation per dimensionless volume flow $\Pi_{Q}/\Pi_{V}$ over dimensionless down-channel pressure gradient $\Pi_{p,z}$ for a power-law exponent of n=0.2 and various screw-pitch ratios $t/D_{b}$.

Another interesting parameter is the dissipation per throughput. This gives an indication of the potential melt temperature increase; for an adiabatic extrusion process with temperature-independent viscosity, this value would be proportional to the melt temperature increase. Figure 12 shows the dimensionless dissipation per throughput over the dimensionless down-channel pressure gradient for a power-law exponent n=0.2 and various screw-pitch ratios. As observed above, the dissipation increases with increasing screw-pitch ratio because the cross-channel flow increases. Above we mentioned that the total dimensionless dissipation is minimal for pure drag flow. The dissipation per throughput, however, is minimal for slightly over ridden melt-conveying zones. In pressure build-up zones, the dissipation per throughput increases significantly with increasing pressure gradient because the throughput decreases and becomes zero, reaching the dam-up pressure. Considering the dissipation per throughput, it is even more obvious that the influence of the screw-pitch ratio vanishes for strongly over ridden screw zones because the pressure-flow component becomes dominating.

## 4. Analytical Approximation

The results presented above provide numerical solutions for the dimensionless throughput $\Pi_{V}$, and dimensionless dissipation $\Pi_{Q}$ depending on the independent dimensionless influencing parameters identified by applying the theory of similarity. Since numerical solutions are time-consuming and require considerable expert knowledge, analytical models that describe the throughput and dissipation as functions of the influencing parameters are usually desired for practical screw design. We have used HeuristicLab [50], an open-source software to derive models describing the relationships of target variables and several independent variables by means of symbolic regression based on genetic programming.

### 4.1. Symbolic Regression—Modeling

In general, symbolic regression searches for a mathematical expression for the relation between the target variable and the influencing variables without specifying the structure of the model. In contrast to symbolic regression, conventional regression requires a predefined model structure and searches for the coefficients that best fit the data. If the preselected model structure does not fit the data well, other functions must be tested until a good model is found. This procedure is time-consuming and the results of the analysis strongly depend on the functions and models tested. Symbolic regression, however, involves finding both the best model structure and its coefficients, optimizing them simultaneously [41,51]. Affenzeller et al. [52] provided fundamental theoretical background on genetic algorithms and genetic programming. Genetic algorithms are population-based metaheuristics; the basic principle is to apply evolutionary concepts from natural evolution. This means that starting with a random initial population of individual models, the models are first evaluated and then selected, and finally a new population of solutions is produced. The most important genetic operators for producing a new population are [52]:

- Parent selection, which means to select parents for “mating” and recombination to generate off-springs that form the next generation. For producing a new generation the two operations crossover and mutation are used.
- Crossover, also called recombination, basically takes two or more different solutions and recombines them to form a new solution.
- Mutation is a random modification that alters one or more solutions. Since mutation can result in a solution that differs completely from the previous solution, it is possible to arrive at even better solutions.
- Replacement is choosing which of the new candidate solutions generated by crossover and mutation operations become members of the next generation and replace some of the old solutions.

Solving a practical problem by using genetic programming requires the following to be specified [51]:

- a terminal set, which is a set of input variables, functions with no argument, and constants;
- a function set, which consists of functions that are used to generate the symbolic regression solution (e.g., arithmetic functions, trigonometric functions, Boolean operations);
- a fitness function, which is a quality measure for evaluating the generated solutions (e.g., mean squared error, mean absolute error);
- algorithm and control parameters, which include control parameters of the algorithm, such as population size, crossover and mutation probability;
- a termination criterion, which is generally the maximum number of generations or a problem-specific success criterion.

**Figure 13:**
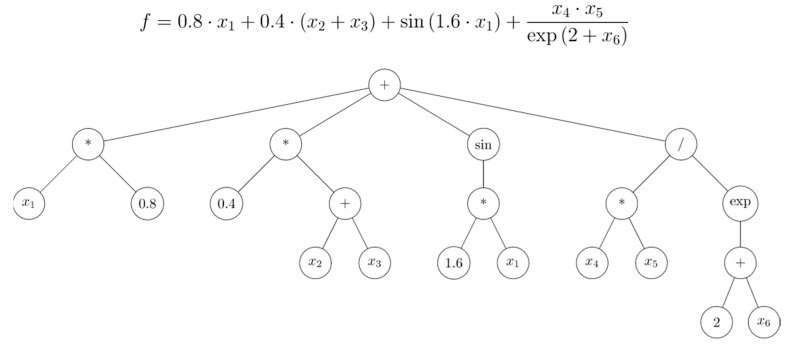
Symbolic regression model example in mathematical notation and in symbolic expression tree form.

The first two specifications basically define the search space for symbolic regression based on genetic programming. If the function set were unrestricted, the search space would theoretically be infinite. Usually, tree-based genetic programming is used, and the symbolic regression solution is given in the form of a symbolic expression tree, as shown in Figure 13. Symbolic regression based on genetic programming is well suited to this problem, because firstly, the underlying model structure is unknown, and secondly, the result is a mathematical expression that can be manipulated and easily implemented into screw-calculation programs.

In our case, the aim was to develop three different analytical equations: one for the dimensionless throughput $\Pi_{V}$ as a function of the dimensionless pressure gradient $\Pi_{p,z}$, the power-law exponent n, and the screw-pitch ratio $t/D_{b}$; one for the dimensionless dissipation $\Pi_{Q}$ as a function of $\Pi_{p,z}$, n, and $t/D_{b}$; and a final one for $\Pi_{Q}$ as a function of $\Pi_{V}$, n, and $t/D_{b}$. Table 6 summarizes the target and influencing variables for the three approximation equations.

The NSGA-II algorithm [53] was used to derive the analytical approximation equations for the throughput model $\Pi_{V}=f\left(\Pi_{p,z},n,t/D_{b}\right)$. For the two dissipation models $\Pi_{Q}=f\left(\Pi_{p,z},n,t/D_{b}\right)$ and $\Pi_{Q}=f\left(\Pi_{V},n,t/D_{b}\right)$, we employed the NSGA-II and OSGA algorithms [54]. The NSGA-II is a multi-objective non-dominated sorting genetic algorithm that simultaneously optimizes model quality and model complexity [55]. The OSGA is the offspring selection genetic algorithm that optimizes model quality only. Aside from being accurate, the models to be obtained had to be simple and interpretable. Thus, we sought the optimal trade-off between high model accuracy and low model complexity. In order to reduce the search space and limit model complexity, we restricted the model size and the function set. The model size was limited to a maximum tree length of 100. For the function set, four different setups were created with different functions and levels of complexity allowed, as listed in Table 7. Note that the argument of more complex functions (square, exponential, logarithm, sine, and cosine) were limited in order to avoid nested functions. To account for statistical variations in the initial population, each of the four settings was repeated 25 times by conducting an experiment, which resulted in a total of 100 models for each target variable.

Model quality was determined by means of the coefficient of determination—Pearson R^2^ (Equation (63)). The coefficient of determination $R^{2}$, with $y_{i}$ as the real values, ${\hat{y}}_{i}$ as the regression values, and $\bar{y}$ as the mean value of the real values, was optimized by the algorithm searching for regression models. The coefficient of determination is in the range between 0 and 1, where $R^{2}=0$ means that the symbolic regression model does not fit the simulation data at all, and $R^{2}=1$ means that the symbolic regression model fits the simulation data perfectly [52].

(63) $$R^{2}=1-\frac{\sum_{i=1}^{N}{\left(y_{i}-{\hat{y}}_{i}\right)}^{2}}{\sum_{i=1}^{N}{\left(y_{i}-\bar{y}\right)}^{2}}.$$

For generating the analytical approximation models, Data Set 1 was used as training set of the algorithms. These models were then analyzed based on Data Sets 1 and 2, and the most promising models were preselected for the subsequent procedure. Combining Data Sets 1 and 2, these preselected models were simplified, after which their constants were further optimized. Finally, for each analytical approximation equation, the best solution was selected by evaluating the model with Data Sets 1 and 2. For the dimensionless throughput model, the coefficient of determination $R^{2}$, the mean absolute error (MAE) (Equation (64)), and the maximum absolute error (Equation (65)) were analyzed. For the dissipation models, the mean relative error (MRE) (Equation (66)) and the maximum relative error (Equation (67)) were analyzed in addition.

(64) $$MAE=\frac{1}{N}\overset{N}{\underset{i=1}{\sum }}\left|y_{i}-{\hat{y}}_{i}\right|.$$

(65) $$e_{max}=\max\left(\left|y_{i}-{\hat{y}}_{i}\right|\right).$$

(66) $$MRE=\frac{1}{N}\overset{N}{\underset{i=1}{\sum }}\frac{\left|y_{i}-{\hat{y}}_{i}\right|}{y_{i}}.$$

(67) $$RE_{max}=\max\left(\frac{\left|y_{i}-{\hat{y}}_{i}\right|}{y_{i}}\right).$$

### 4.2. Symbolic Regression—Results

We derived three different symbolic regression results by modeling the 3690 design points of Data Set 1 and by additionally using the 8235 design points of Data Set 2 for the final optimization and selection procedure. One model was developed for the dimensionless throughput $\Pi_{V}$, and two models were developed for the dimensionless dissipation $\Pi_{Q}$.

#### 4.2.1. Dimensionless Throughput $\Pi_{V}$

Our analytical approximation equation predicting the dimensionless throughput $\Pi_{V}$ as a function of $\Pi_{V}=f\left(\Pi_{p,z},n,t/D_{b}\right)$ is given by Equation (68), with the sub-functions $A_{1}$ to $A_{6}$ containing 32 coefficients. The sub-functions and the corresponding coefficients are given in the Appendix A.1 in Equations (74) to (79) and Table A1, respectively. This developed model predicts the two-dimensional throughput-pressure relationship of non-Newtonian fluids for pressure-generating and-pressure consuming metering channels, including strongly over-ridden screw zones. The overall model structure is relatively simple, consisting only of arithmetic operations and sine and cosine functions. Since the model has no nested functions, and as the arguments of sine and cosine are constants and variables only, mathematical manipulations such as differentiation are easily possible. Additionally, the model is easy to implement in a simulation program, and fast computation can be achieved.

(68) $$\Pi_{V}\left(\Pi_{p,z},n,t/D_{b}\right)=A_{1}+A_{2}+\frac{1}{A_{3}+A_{4}}+A_{5}A_{6}.$$

**Figure 14:**
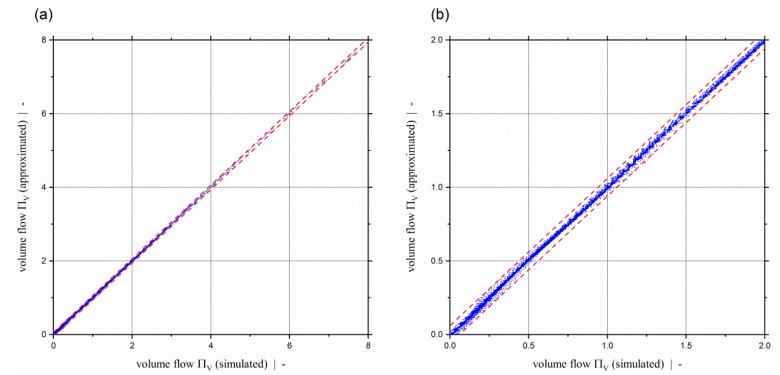
Scatter plot of the dimensionless throughput model $\Pi_{V}=f\left(\Pi_{p,z},n,t/D_{b}\right)$ (**a**) for Data Set 1 and (**b**) for Data Set 2. The dashed red lines indicate an absolute error of 0.06.

**Figure 15:**
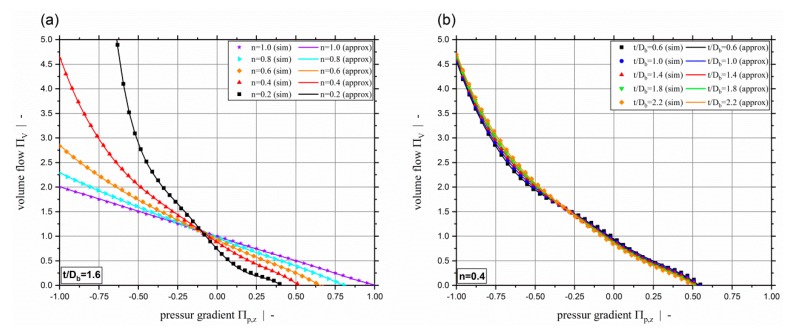
Comparison of the approximation model for the dimensionless flow rate $\Pi_{V}$ as a function of $\Pi_{p,z}$, n, and $t/D_{b}$, with the simulation results for various power law exponents and a screw-pitch ratio of $t/D_{b}=1.6$ (**a**), and for various screw-pitch ratios and a power-law exponent of n=0.4 (**b**).

The accuracy of the model was evaluated based on an error analysis. To this end, the coefficient of determination, the mean absolute error, and the maximum absolute error were evaluated for both data sets: Data Set 1 was used for training the model, and Data Set 2 was additionally used for model preselection and final optimization. The results of the error analysis are listed in Table 8. With this model, a coefficient of determination of $R^{2}=0.999599$ and above can be achieved, the mean absolute error is in the range of MAE≈0.01, and the maximum absolute error is $e_{max}=0.15169$ and $e_{max}=0.088444$, respectively, for Data Sets 1 and 2. Note that the maximum error $e_{max}=0.15169$ for Data Set 1 represents a data point for slightly negative dimensionless throughputs. Excluding the design points that are beyond the limit of negative throughputs ($\Pi_{V}\leq 0$) shifts the maximum absolute error for Data Set 1 to $e_{max}^{*}=0.086905$, which is in the same range as that for Data Set 2. Scatter plots showing the approximated solutions versus the exact numerical solutions for Data Sets 1 and 2 are given in Figure 14a,b, respectively. The dashed lines indicate an absolute error of 0.06. The scatter plot illustrates the excellent accuracy of the presented model. A comparison of the dimensionless throughput pressure model with the numerical simulation results is given by Figure 15 in the form of dimensionless throughput-pressure curves. Again, it can be seen that the extended approximation model for $\Pi_{V}=f\left(\Pi_{p,z},n,t/D_{b}\right)$ fits the simulation results really accurately.

#### 4.2.2. Dimensionless Dissipation $\Pi_{Q}$

We developed two analytical approximation equations that predict the dimensionless dissipation $\Pi_{Q}$ of a two-dimensional flow of power-law fluids in metering channels. Equation (69) predicts the dimensionless dissipation $\Pi_{Q}$ as a function of $\Pi_{Q}=f\left(\Pi_{p,z},n,t/D_{b}\right)$, and Equation (70) as a function of $\Pi_{Q}=f\left(\Pi_{V},n,t/D_{b}\right)$. $B_{1}$ to $B_{10}$ are the sub-functions of $\Pi_{Q}=f\left(\Pi_{p,z},n,t/D_{b}\right)$, which contain a total number of 45 coefficients, as given in the Appendix A.2 by Equations (80) to (89) and Table A2, respectively. $C_{1}$ to $C_{8}$ are the sub-functions of $\Pi_{Q}=f\left(\Pi_{V},n,t/D_{b}\right)$ and contain a total number of 44 coefficients, as given in the Appendix A.3 by Equations (90) to (97) and Table A3, respectively. Analogously to the throughput models, the overall model structure is also relatively simple for the two dissipation models, as they consist only of arithmetic operations and exponential and logarithmic functions. Again, no nested functions are present, and the arguments of the exponential and logarithmic functions consist of constants and variables only. This enables easy mathematical manipulation and implementation, as well as fast computation of the dissipation models.

(69) $$\Pi_{Q}\left(\Pi_{p,z},n,t/D_{b}\right)=B_{1}+B_{2}\left(B_{3}+B_{4}\right)+B_{5}B_{6}\left(B_{7}+\frac{B_{8}}{B_{9}+B_{10}}\right).$$

(70) $$\Pi_{Q}\left(\Pi_{V},n,t/D_{b}\right)=C_{1}+C_{2}\left(C_{3}+\frac{C_{4}}{C_{5}}+\frac{C_{6}}{C_{7}+C_{8}}\right).$$

**Figure 16:**
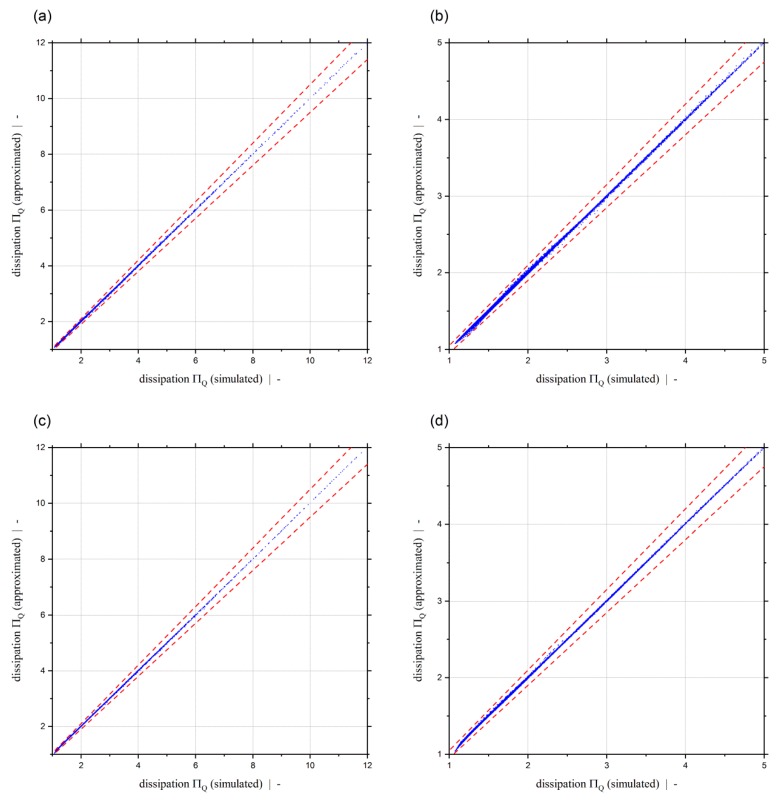
Scatter plots of the dimensionless dissipation models: $\Pi_{Q}=f\left(\Pi_{p,z},n,t/D_{b}\right)$ (**a**) for Data Set 1, (**b**) for Data Set 2; $\Pi_{Q}=f\left(\Pi_{V},n,t/D_{b}\right)$ (**c**) for Data Set 1, (**d**) for Data Set 2. The dashed red lines indicate a relative error of ±5%.

Our dissipation models predict the two-dimensional dissipation of non-Newtonian fluids for pressure-generating and pressure-consuming metering channels, including strongly overridden screw zones. The effect of the cross-channel flow, which has a significant impact on the viscous dissipation, is accounted for by the screw-pitch ratio $t/D_{b}$. Predictions are possible for either a given pressure-gradient or a given throughput. Both viscous dissipation models exhibit outstanding accuracy, as shown by an error analysis using Data Sets 1 and 2, the results of which are listed in Table 9. Mean relative errors of MRE≤0.4% and MRE≤0.27% are achieved by the dissipation models $\Pi_{Q}=f\left(\Pi_{p,z},n,t/D_{b}\right)$ and $\Pi_{Q}=f\left(\Pi_{V},n,t/D_{b}\right)$, respectively. The maximum error of both models is $RE_{max}=4.17\%$, and thus significantly lower than 5%. The excellent quality of the dissipation models is also highlighted by scatter plots showing the approximated dissipation versus the corresponding simulation results in Figure 16. The dashed lines indicate a maximum relative error of ±5%, and, as confirmed by the error analysis, it can be seen that all data points are clearly within ±5%.

**Figure 17:**
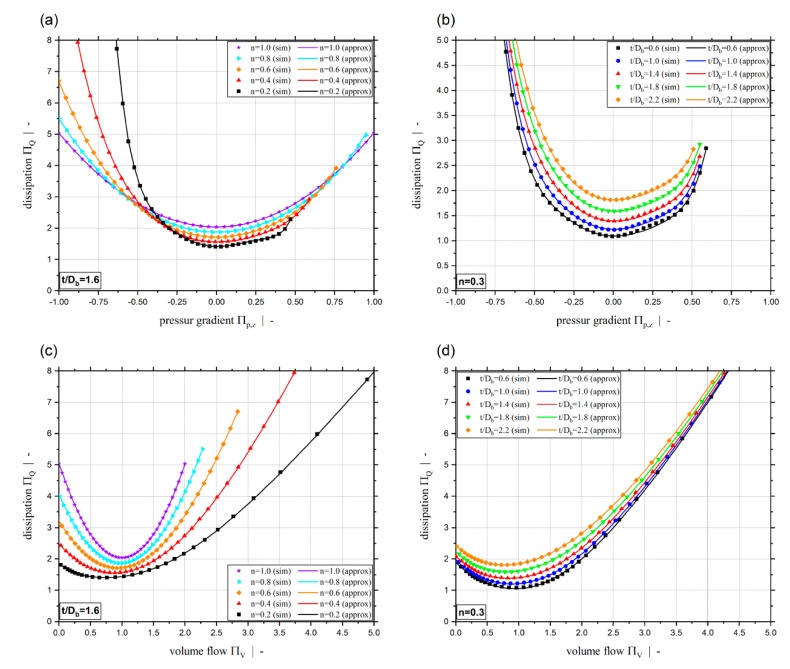
Comparison of approximation models for the dimensionless dissipation $\Pi_{Q}$ with the simulation results for: (**a**) $\Pi_{Q}=f\left(\Pi_{p,z},n,t/D_{b}\right)$ for various power-law exponents and $t/D_{b}=1.6$; (**b**) $\Pi_{Q}=f\left(\Pi_{p,z},n,t/D_{b}\right)$ for various screw-pitch ratios and n=0.3; (**c**) $\Pi_{Q}=f\left(\Pi_{V},n,t/D_{b}\right)$ for various power-law exponents and $t/D_{b}=1.6$; and (**d**) $\Pi_{Q}=f\left(\Pi_{V},n,t/D_{b}\right)$ for various screw-pitch ratios and n=0.3.

For both dissipation models developed, Figure 17 shows a comparison between approximated and simulated dissipation for a constant screw-pitch ratio and various power-law exponents (a), (c), and for constant power-law exponent and different screw pitch ratios (b), (d). Again, it can be seen that the results of both extended approximation models $\Pi_{Q}=f\left(\Pi_{p,z},n,t/D_{b}\right)$ and $\Pi_{Q}=f\left(\Pi_{V},n,t/D_{b}\right)$ fit the simulation values really accurately.

## 5. Validation

To obtain a more objective and unbiased assessment of the accuracy of our models, we tested them on a data set that had not been used for training, model selection, and model optimization—Data Set 3. Variation of the dimensionless influencing parameters for Data Set 3, described above and listed in Table 3, resulted in a total of 927 design points for the final model evaluation. The parameter range was within the scope of validity defined by Data Set 1. The values of the dimensionless down-channel pressure gradient $\Pi_{p,z}$, the power-law exponent n, and the screw-pitch ratio $t/D_{b}$ were defined randomly and differ from those used for training and optimizing the models. The results of an error analysis based on Data Set 3 are listed in Table 10. With the throughput model $\Pi_{V}=f\left(\Pi_{p,z},n,t/D_{b}\right)$, a coefficient of determination of $R^{2}=0.999825$ and a mean absolute error of MAE=0.013374 is achieved, which indicates excellent accuracy of the model. Note that the maximum absolute error $e_{max}=0.231581$ is equivalent to a relative error RE=1.819%. With the dissipation models, mean relative errors MRE≤0.4% and maximum relative errors $RE_{max}\leq 4.66\%$ are achieved, which also indicates excellent accuracy. All three models exhibit very good performance on the independent evaluation data set.

**Figure 18:**
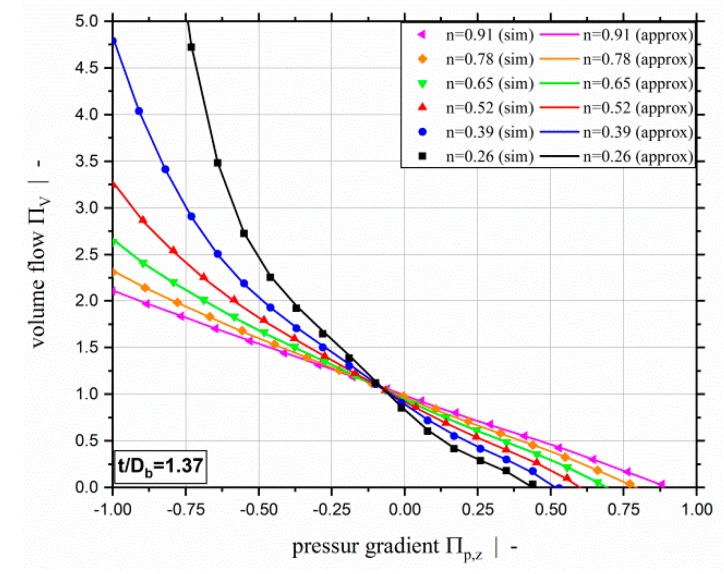
Comparison of approximation model for the dimensionless volume flow $\Pi_{V}$ over dimensionless down-channel pressure gradient $\Pi_{p,z}$ of a two-dimensional flow, for a screw-pitch ratio of $t/D_{b}=1.37$ and various power-law exponents based on the evaluation data set.

**Figure 19:**
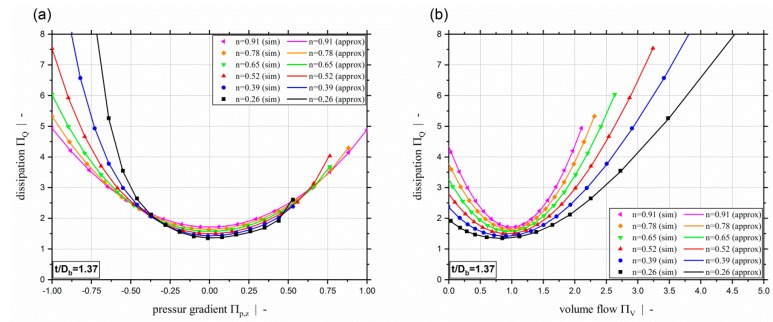
Comparison of approximation models for the dimensionless dissipation $\Pi_{Q}$ as a function of the dimensionless influencing parameters for $t/D_{b}=1.37$ and various power-law exponents based on the evaluation data set. The approximation model $\Pi_{Q}=f\left(\Pi_{p,z},n,t/D_{b}\right)$ is shown in (**a**), and $\Pi_{Q}=f\left(\Pi_{V},n,t/D_{b}\right)$ is shown in (**b**).

Figure 18 compares the simulated and approximated throughput-pressure relationships for the power-law exponents chosen for the evaluation set and a screw-pitch ratio of $t/D_{b}=1.37$. In Figure 19, a comparison of simulation and approximation is given for the dissipation based on the evaluation data set for various power-law exponents and a screw-pitch ratio of $t/D_{b}=1.37$. Figure 19a shows the approximation model $\Pi_{Q}=f\left(\Pi_{p,z},n,t/D_{b}\right)$, and Figure 19b the approximation model $\Pi_{Q}=f\left(\Pi_{V},n,t/D_{b}\right)$. As confirmed by the error analysis, excellent agreement with the simulation results can be observed for all three models. Our extended symbolic regression models for predicting the pumping capability and viscous dissipation of two-dimensional flows in single-screw extrusion are, therefore, highly accurate, not only on the training data but on independent evaluation data within the complete range of application. This means that accurate predictions are guaranteed within the complete range of validity. Note that the range of validity is defined by the ranges of the dimensionless influencing parameters of the parametric design study (see Table 1). The broad scope of the parametric design study, however, covers almost all polymer melts and processing conditions. Moreover, it covers virtually all screw designs, including general-purpose screws and special screw designs, such as wave and energy-transfer screws.

## 6. Conclusions

We have presented three generalized symbolic regression models for predicting the two-dimensional polymer melt flow in the metering channel of a single-screw extruder:

1. $\Pi_{V}$ as a function of $\Pi_{p,z}$, n, and $t/D_{b}$;
2. $\Pi_{Q}$ as a function of $\Pi_{p,z}$, n, and $t/D_{b}$; and
3. $\Pi_{Q}$ as a function of $\Pi_{V}$, n, and $t/D_{b}$.

These models take into account the effect of the non-Newtonian fluid behavior and cross-channel flow, covering an extended range of applications, including strongly over-ridden screw zones. Hence, our models apply for both general purpose screws, as well as special screw designs, like wave and energy-transfer screws, covering almost all possible processing conditions. By applying the theory of similarity, we identified a set of independent dimensionless influencing parameters that fully describe the two-dimensional flow of polymer melts in metering channels: the dimensionless pressure gradient $\Pi_{p,z}$, the power-law exponent n, and the screw-pitch ratio $t/D_{b}$. Due to the relationship between the dimensionless flow rate $\Pi_{V}$ and the dimensionless pressure-gradient $\Pi_{p,z}$, it was possible to identify a second, different set of independent dimensionless influencing parameters for the viscous dissipation: the dimensionless flow rate $\Pi_{V}$, the power-law exponent n, and the screw-pitch ratio $t/D_{b}$. Based on the findings of our dimensional analysis, an extensive parametric design study was carried out, which resulted in numerical solutions for throughput and dissipation. The ranges of the dimensionless influencing parameters were chosen such that they cover almost all polymer materials and processing conditions occurring in polymer extrusion. Unlike with existing models, the lower limit of the dimensionless down-channel pressure gradient was not limited to achieve approximately $\Pi_{V}\leq 2.0$, but was fixed to $\Pi_{p,z,min}=-1.0$. Screw calculations have shown that for special screw designs, such as wave and energy-transfer screws, and for polymer melts with distinct shear-thinning behavior, the lower limit for the dimensionless down-channel pressure gradient of previous models is insufficient. Additionally, we extended the range for the screw-pitch ratio significantly. Our approximation equations were derived by applying symbolic regression based on genetic programming. All three models are continuous over the whole range of applications, simple, and contain a moderate number of coefficients. In contrast to previously presented approximation equations for the throughput-pressure relationship, our model has no complex and deeply nested functions of sine, cosine, exponential, and logarithmic functions. Rather, the arguments of these functions are combinations of constants and variables only. This is also the case for the dissipation models. Additionally, the presented models accurately approximate the numerical solutions, as proven by means of an independent and unbiased evaluation data set. The throughput model yields a coefficient of determination $R^{2}=0.999825$ and a mean absolute error MAE=0.013374. The dissipation models yield a coefficient of determination $R^{2}\geq 0.999845$, a mean relative error MRE≤0.4%, and a maximum relative error $RE_{max}\leq 4.66\%$.

Performing minor adaptions of the boundary conditions, our models are additionally applicable to injection molding. To this end, the velocity boundary conditions have to be rewritten as

(71) $$v_{1,z}=v_{b}\cos\left(\varphi_{b}\right)+\dot{s}\sin\left(\varphi_{b}\right),$$

(72) $$v_{1,x}=v_{b}\sin\left(\varphi_{b}\right)-\dot{s}\cos\left(\varphi_{b}\right),$$

with the axial retraction speed $\dot{s}$. The dimensionless parameters are then related to these velocity boundary conditions and the screw-pitch ratio has to be adapted according to

(73) $$\tan\left(\varphi_{1}\right)=\frac{v_{1,x}}{v_{1,z}}.$$

Our models have a simple structure and can be implemented easily in screw-calculation routines or any other application. Implementations of pumping models in screw-calculation routines are shown by [56,57], which also cover solids conveying and melting. Melt-conveying, however, is one of the most critical functional zones. Moreover, easy mathematical operations, like forming the derivative, are possible. Additionally, our models enable fast and stable prediction of both the throughput-pressure relationship and viscous dissipation without the need for complex, time-consuming, and computationally expensive numerical procedures, which require lots of expertise knowledge. Although these models are not capable of outperforming numerical simulations in terms of accuracy, as they approximate the numerical solutions for a large search space, they are considerably faster than solving the flow equations numerically. Since viscous dissipation is mainly responsible for the axial melt temperature increase, our models for the dissipation provide an excellent basis for calculating the axial melt-temperature development. Combining the models for the throughput-pressure relationship and the viscous dissipation allows the effect of the temperature-dependent viscosity on the axial pressure profile to be considered. This means non-isothermal throughput-pressure calculations are possible. In addition, for model-based extrusion control, accurate predictions of the extrusion characteristics are necessary, like the dissipated energy is important for multi-zone temperature control. Note that for screw calculations the dimensionless influencing parameters must first be determined and then the dimensionless target variables calculated, which must finally be transformed back to the dimensional representation.

## Figures and Tables

**Table 1:** Ranges of variation for $t/D_{b}$, n, and $\Pi_{p,z}$ for Data Set 1.

Variable	Min	Max	Increment
$t/D_{b}$	0.6	2.4	0.2
n	0.2	1.0	0.1
$\Pi_{p,z}$	−1.0	var. ($\Pi_{V,min}\leq 0$)	var.

**Table 2:** Ranges of variation for $t/D_{b}$, n, and $\Pi_{p,z}$ for Data Set 2.

Variable	Min	Max	Increment
$t/D_{b}$	0.6	2.0	0.1
n	0.2	1.0	0.1
$\Pi_{p,z}$	var. ($\Pi_{V,max}\geq 2.0$)	var. ($\Pi_{V,min}\leq 0$)	var.

**Table 3:** Values for $t/D_{b}$, n, and $\Pi_{p,z}$ for Data Set 3.

$t/D_{b}$	0.62	0.88	1.29	1.31	1.37	1.61	1.83	2.19	2.35
n	0.26	0.39	0.52	0.65	0.78	0.91			
$\Pi_{p,z}$	17 equidistant divisions								

**Table 4:** Summary of variation for $t/D_{b}$, n, and $\Pi_{p,z}$ for all three data sets.

Data Set	$\mathrm{Quantity}\;t/D_{b}$	Quantity n	$\mathrm{Quantity}\;\Pi_{p,z}$	Total Number
1	10	9	41	3690
2	15	9	61	8235
3	9	6	18	972

**Table 5:** Parameter setup chosen for the numerical simulations.

Parameter	Value
number of nodes	1001
abortion criterion	$\Delta \Pi_{V}<10^{-8}$
variation of $C_{1}$ for linearization	0.01
variation of $C_{2}$ for linearization	0.01
variation of $\Pi_{p,x}$ for linearization	0.01
max. dimensionless viscosity $\eta^{*}$	$10^{3}$
min. dimensionless viscosity $\eta^{*}$	$10^{-3}$

**Table 6:** Target and influencing variables for the analytical approximation equations.

Target Variable	Influencing Variables
$\Pi_{V}$	$\Pi_{p,z}$, n, $t/D_{b}$
$\Pi_{Q}$	$\Pi_{p,z}$, n, $t/D_{b}$
$\Pi_{Q}$	$\Pi_{V}$, n, $t/D_{b}$

**Table 7:** Mathematical building blocks for the analytical approximation—four different setups.

Tree grammar Variant	(a)	(b)	(c)	(d)
**Constant**	✓	✓	✓	✓
**State variable**	✓	✓	✓	✓
**Addition**	✓	✓	✓	✓
**Multiplication**	✓	✓	✓	✓
**Division**	✓	✓	✓	✓
**Square**	✓	✓	✓	✓
**Exponential**		✓		✓
**Logarithm**		✓		✓
**Sine**			✓	✓
**Cosine**			✓	✓

**Table 8:** Results of the error analysis of the approximation model $\Pi_{V}=f\left(\Pi_{p,z},n,t/D_{b}\right)$.

Quality Measure	Unit	Data Set 1	Data Set 2
Pearson’s R^2^	-	0.999974	0.999599
mean abs. error	-	0.011689	0.009782
max. abs. error	-	0.151690	0.088444

**Table 9:** Results of the error analysis for the approximation model $\Pi_{Q}=f\left(\Pi_{p,z},n,t/D_{b}\right)$ and $\Pi_{Q}=f\left(\Pi_{V},n,t/D_{b}\right)$.

Quality Measure	Unit	$\Pi_{Q}=f\left(\Pi_{p,z},n,t/D_{b}\right)$		$\Pi_{Q}=f\left(\Pi_{V},n,t/D_{b}\right)$	
		Data Set 1	Data Set 2	Data Set 1	Data Set 2
Pearson’s R^2^	-	0.999997	0.999830	0.999999	0.999934
mean abs. error	-	0.009385	0.007797	0.006564	0.004990
max. abs. error	-	0.109750	0.082440	0.084250	0.043206
mean rel. error	%	0.3571	0.4031	0.2415	0.2696
max. rel. error	%	4.169	4.170	3.530	3.521

**Table 10:** Evaluation results of the error analysis for the approximation models.

Quality Measure	Unit	$\Pi_{V}\left(\Pi_{p,z},n,t/D_{b}\right)$	$\Pi_{Q}\left(\Pi_{p,z},n,t/D_{b}\right)$	$\Pi_{Q}\left(\Pi_{V},n,t/D_{b}\right)$
Pearson’s $R^{2}$	-	0.999825	0.999845	0.999978
mean abs. error	-	0.013374	0.022295	0.008686
max. abs. error	-	0.231581	1.07895	0.226912
mean rel. error	%		0.4056	0.2470
max. rel. error	%		3.976	4.663

## Nomenclature

Symbol	Parameter	Symbol	Parameter
A_1_–A_6_	sub-functions	ϱ	melt density
B_1_–B_10_	sub-functions	p	hydrostatic pressure
C_1_–C_68_	sub-functions	D	rate-of-deformation tensor
a_00_–a_31_	constants	L	velocity gradient tensor
b_00_–b_44_	constants	τ	viscous stress tensor
c_00_–c_43_	constants	τ_ij_	components of the stress tensor
D_b_	barrel diameter	$\dot{V}$	volumetric flow rate
h	channel height	${\dot{q}}_{diss}$	specific viscous dissipation
w	channel width	${\dot{Q}}_{diss}$	viscous dissipation
i	number of parallel screw channels	ξ	dimensionless coordinate up-channel direction
e	flight width	ν_z_, ν_x_	dimensionless velocities
δ	flight clearance	${\dot{\gamma }}^{*}$	dimensionless shear rate
t	screw pitch	$\eta^{*}$	dimensionless viscosity
$\varphi_{b}$	helix angle at outer diameter	τ_ij_^*^	dimensionless components of the stress tensor
v_b_	barrel velocity	$\Pi_{p,z}$, $\Pi_{p,x}$	dimensionless pressure gradients
v_b,x_	barrel velocity in z-direction	$\Pi_{V}$	dimensionless volumetric flow rate
v_b,z_	barrel velocity in x-direction	$\pi_{q}$	dimensionless specific viscous dissipation
x, y, z	spatial coordinates	$\Pi_{Q}$	dimensionless viscous dissipation
v	velocity vector	J	Jacobi matrix
v_x_, v_y_, v_z_	velocity components	R^2^	coefficient of determination
N	screw speed	MAE	mean absolute error
η	viscosity	$e_{max}$	maximum absolute error
$\dot{\gamma }$	shear rate	MRE	mean relative error
K	consistency	$RE_{max}$	maximum relative error
n	power-law exponent		

## Appendix A

### Appendix A.1. Sub-Functions of Equation (68): $\Pi_{V}=f\left(\Pi_{p,z},n,t/D_{b}\right)$

(74) $$A_{1}=a_{00}+a_{01}n+a_{02}\Pi_{p,z}+a_{03}\sin\left(a_{04}+a_{05}\Pi_{p,z}\right).$$

(75) $$A_{2}=\frac{\Pi_{p,z}}{a_{06}+a_{07}n+a_{08}\Pi_{p,z}+a_{09}t/D_{b}+\frac{1}{a_{10}+a_{11}n+a_{12}\sin\left(a_{13}\Pi_{p,z}\right)}}.$$

(76) $$A_{3}=a_{14}+a_{15}n+a_{16}\Pi_{p,z}+a_{17}t/D_{b}.$$

(77) $$A_{4}=\frac{a_{18}\Pi_{p,z}}{a_{19}+a_{20}n+a_{21}\Pi_{p,z}+a_{22}t/D_{b}+\sin\left(a_{23}\Pi_{p,z}\right)}.$$

(78) $$A_{5}=\frac{\cos\left(a_{24}\Pi_{p,z}\right)}{n^{2}}.$$

(79) $$A_{6}=a_{25}+a_{26}\Pi_{p,z}+\frac{a_{27}\Pi_{p,z}^{3}}{n+a_{28}\Pi_{p,z}}+a_{29}t/D_{b}+a_{30}\sin\left(a_{31}\Pi_{p,z}\right).$$

**Table A1:** Rounded values of the constants for $\Pi_{V}=f\left(\Pi_{p,z},n,t/D_{b}\right)$.

Constant	Value	Constant	Value	Constant	Value
a_00_	−0.12014	a_11_	−3.4056	a_22_	0.18277
a_01_	0.25392	a_12_	−26.252	a_23_	2.6134
a_02_	2.0905	a_13_	1.5616	a_24_	3.2436
a_03_	3.2395	a_14_	2.0161	a_25_	−0.0017916
a_04_	2.9348	a_15_	2.1787	a_26_	0.078972
a_05_	0.70199	a_16_	0.61167	a_27_	0.066612
a_06_	−0.050140	a_17_	0.42387	a_28_	0.14623
a_07_	−0.95819	a_18_	−5.8881	a_29_	−0.0033694
a_08_	−0.32345	a_19_	0.77710	a_30_	−0.019723
a_09_	0.0014903	a_20_	3.1672	a_31_	4.1031
a_10_	−27.930	a_21_	2.8402		

### Appendix A.2. Sub-Functions of Equation (69): $\Pi_{Q}=f\left(\Pi_{p,z},n,t/D_{b}\right)$

(80) $$B_{1}=b_{00}+b_{01}n+b_{02}\Pi_{p,z}^{2}+b_{03}{\left(n+b_{04}t/D_{b}\right)}^{2}.$$

(81) $$B_{2}=b_{05}+b_{06}n.$$

(82) $$B_{3}=b_{07}n^{2}+b_{08}\Pi_{p,z}+n\left[b_{09}+\Pi_{p,z}\left(b_{10}+b_{11}t/D_{b}\right)+b_{12}t/D_{b}\right].$$

(83) $$B_{4}=b_{13}\Pi_{p,z}^{2}t/D_{b}+b_{14}t/D_{b}+b_{15}t/{D_{b}}^{2}.$$

(84) $$B_{5}=e^{b_{16}n}\Pi_{p,z}^{2}\left(b_{17}+b_{18}n\right)\left(b_{19}+b_{20}\Pi_{p,z}\right).$$

(85) $$B_{6}=e^{b_{21}n}+b_{22}+b_{23}n+b_{24}\Pi_{p,z}+b_{25}\Pi_{p,z}^{2}.$$

(86) $$B_{7}=b_{26}+e^{b_{27}n}+e^{b_{28}\Pi_{p,z}}\left(b_{29}+b_{30}n\right)+b_{31}\Pi_{p,z}+b_{32}t/D_{b}.$$

(87) $$B_{8}=b_{33}+b_{34}t/D_{b}.$$

(88) $$B_{9}=b_{35}+e^{b_{36}n}+e^{b_{37}n+b_{38}\Pi_{p,z}}+b_{39}e^{b_{40}\Pi_{p,z}}.$$

(89) $$B_{10}=b_{41}n+b_{42}n^{2}+b_{43}\Pi_{p,z}^{2}+b_{44}t/{D_{b}}^{2}.$$

**Table A2:** Rounded values of the constants for $\Pi_{Q}=f\left(\Pi_{p,z},n,t/D_{b}\right)$.

Constant	Value	Constant	Value	Constant	Value
b_00_	0.86172	b_15_	−0.26376	b_30_	−11.314
b_01_	0.10200	b_16_	−24.303	b_31_	−10.082
b_02_	3.0156	b_17_	−0.25811	b_32_	−0.052856
b_03_	0.0014701	b_18_	0.26056	b_33_	−393.64
b_04_	16.281	b_19_	−7.1580	b_34_	−90.030
b_05_	1.6249	b_20_	16.080	b_35_	−68.179
b_06_	−1.6156	b_21_	18.452	b_36_	4.8797
b_07_	−0.0099142	b_22_	98.805	b_37_	−14.870
b_08_	−0.10634	b_23_	−136.28	b_38_	−11.171
b_09_	0.13419	b_24_	356.33	b_39_	190.64
b_10_	0.25265	b_25_	416.31	b_40_	2.1170
b_11_	−0.061868	b_26_	3.2554	b_41_	91.599
b_12_	−0.10227	b_27_	4.6268	b_42_	−224.08
b_13_	0.17363	b_28_	3.1055	b_43_	120.47
b_14_	0.19517	b_29_	1.6456	b_44_	20.069

### Appendix A.3. Sub-Functions of Equation (70): $\Pi_{Q}=f\left(\Pi_{V},n,t/D_{b}\right)$

(90) $$C_{1}=c_{00}+c_{01}n+c_{02}\Pi_{V}+c_{03}t/D_{b}.$$

(91) $$C_{2}=e^{c_{04}n}.$$

(92) $$C_{3}=c_{05}+c_{06}n+c_{07}\Pi_{V}+c_{08}t/D_{b}+c_{09}t/{D_{b}}^{2}+c_{10}\ln\left(c_{11}+c_{12}\Pi_{V}\right).$$

(93) $$C_{4}=e^{c_{13}n}\left(c_{14}e^{c_{15}t/D_{b}}+c_{16}n+c_{17}\Pi_{V}+c_{18}\Pi_{V}^{2}+c_{19}t/{D_{b}}^{2}\right).$$

(94) $$C_{5}=c_{20}+e^{c_{21}n+c_{22}\Pi_{V}}+c_{23}n+c_{24}\Pi_{V}+c_{25}\Pi_{V}^{2}+c_{26}t/D_{b}+c_{27}t/{D_{b}}^{2}.$$

(95) $$C_{6}=c_{28}+c_{29}e^{c_{30}\Pi_{V}}+c_{31}\Pi_{V}+c_{32}\Pi_{V}^{2}+c_{33}t/{D_{b}}^{2}.$$

(96) $$C_{7}=c_{34}+e^{c_{35}n}+e^{c_{36}\Pi_{V}}+c_{37}n^{2}+c_{38}\Pi_{V}+c_{39}t/D_{b}+c_{40}t/{D_{b}}^{2}.$$

(97) $$C_{8}=\frac{c_{41}\Pi_{V}}{n\ln\left(c_{42}+c_{43}\Pi_{V}\right)}.$$

**Table A3:** Rounded values of the constants for $\Pi_{Q}=f\left(\Pi_{V},n,t/D_{b}\right)$.

Constant	Value	Constant	Value	Constant	Value
c_00_	−4.0149	c_15_	0.19534	c_30_	−5.7695
c_01_	−0.77962	c_16_	6.1094	c_31_	−2.9650
c_02_	1.4469	c_17_	−11.854	c_32_	2.0976
c_03_	0.11684	c_18_	10.1740	c_33_	0.18684
c_04_	1.7719	c_19_	4.0632	c_34_	1.2438
c_05_	−8.4896	c_20_	5.7393	c_35_	−0.10144
c_06_	0.76173	c_21_	1.3311	c_36_	−3.5935
c_07_	−0.45230	c_22_	−1.4480	c_37_	0.69822
c_08_	−0.14219	c_23_	0.92724	c_38_	−0.029327
c_09_	0.017573	c_24_	0.27244	c_39_	−0.061845
c_10_	2.6071	c_25_	1.8007	c_40_	0.011676
c_11_	17.055	c_26_	1.2338	c_41_	0.85656
c_12_	−0.25102	c_27_	0.83026	c_42_	4.2487
c_13_	−0.87942	c_28_	−0.68791	c_43_	0.64400
c_14_	42.781	c_29_	0.28069		

## References

[1] Rowell H.S., Finlayson D. (1922). Screw Viscosity Pumps. Engineering.

[2] Rowell H.S., Finlayson D. (1928). Screw Viscosity Pumps. Engineering.

[3] Carley J.F., Mallouk R.S., McKelvey J.M. (1953). Simplified Flow Theory for Screw Extruders. Ind. Eng. Chem..

[4] Mallouk R.S., McKelvey J.M. (1953). Power Requirements of Melt Extruders. Ind. Eng. Chem..

[5] Mohr W.D., Saxon R.L., Jepson C.H. (1957). Theory of Mixing in Single-Screw Extruder. Ind. Eng. Chem..

[6] Mohr W.D., Mallouk R.S. (1959). Flow, Power Requirement, and Pressure Distribution of Fluid in a Screw Extruder. Ind. Eng. Chem..

[7] McKelvey J.M. (1953). Experimental Studies of Melt Extrusion. Ind. Eng. Chem..

[8] Rauwendaal C. (2014). Polymer Extrusion.

[9] Rotem Z., Shinnar R. (1961). Non-newtonian flow between parallel boundaries in linear movement. Chem. Eng. Sci..

[10] Krüger H. (1963). Extruder für nicht-newtonsche Schmelzen–Analyse und Vorausberechnung des Betriebsverhaltens. Kunststoffe.

[11] Kroesser F.W., Middleman S. (1965). The Calculation of Screw Characteristics for the Extrusion of non-Newtonian Melts. Polym. Eng. Sci..

[12] Tadmor Z., Gogos Z.G. (2006). Principles of Polymer Processing.

[13] Griffith R.M. (1962). Fully Developed Flow in Screw Extruders. Ind. Eng. Chem. Fund..

[14] Zamodits H.J., Pearson J.R.A. (1969). Flow of Polymer Melts in Extruders. Part I. The Effect of Transverse Flow and of a Superposed Steady Temperature Profile. J. Rheol..

[15] Stellar R.M. (1990). Theoretical Model for Flow of Polymer Melts in the Screw Channel. Polym. Eng. Sci..

[16] Booy M.L. (1981). The Influence of Non-Newtonian Flow on Effective Viscosity and Channel Efficiency in Screw Pumps. Polym. Eng. Sci..

[17] Tadmor Z., Klein I. (1970). Engineering Principles of Plasticating Extrusion.

[18] Fenner R.T. (1977). Developments in the analysis of steady screw extrusion of polymers. Polymer.

[19] Rauwendall C. (1986). Throughput-Pressure Relationship for Power Law Fluids in Single Screw Extruders. Polym. Eng. Sci..

[20] Potente H. (1981). Auslegung von Schmelzeextrudern für Kunststoffschmelzen mit Potenzgesetzverhalten. Kunststoffe.

[21] Potente H. (1983). Approximationsgleichungen für Schmelzeextruder. Rheol. Acta.

[22] Effen N. (1996). Theoretische und experimentelle Untersuchungen zur rechnergestützten Auslegung und Optimierung von Spritzgießplastifiziereinheiten. Ph.D. Thesis.

[23] White J.L., Potente H. (2001). Screw Extrusion.

[24] Pachner S., Loew-Baselli B., Affenzeller M., Miethlinger J. (2017). A Generalized 2D Output Model of Polymer Melt Flow in Single-Screw Extrusion. Int. Polym. Process..

[25] Spalding M.A., Campbell G.A. (2011). An Engineering Approach to the Correction of Rotational Flow Calculations for Single-Screw Extruders—Equation Correction. SPE ANTEC Tech. Pap..

[26] Campbell G.A. (2013). Analyzing and Troubleshooting Single-Screw Extruders.

[27] Kim S.J., Kwon T.H. (1995). A Simple Approach to Determining Three-Dimensional Screw Characteristics in the Metering Zone of Extrusion Processes Using a Total Shape Factor. Polym. Eng. Sci..

[28] Marschik C., Roland W., Loew-Baselli B., Miethlinger J. (2017). Modeling Three-Dimensional Non-Newtonian Flows in Single-Screw Extruders. SPE ANTEC Tech. Pap..

[29] Marschik C., Roland W., Loew-Baselli B., Miethlinger J. (2017). A heuristic method for modeling three-dimensional non-Newtonian flows of polymer melts in single-screw extruders. J. Non Newt. Fluid Mech..

[30] Marschik C., Roland W., Osswald T., Loew-Baselli B., Miethlinger J. (2018). A Heuristic Model for Predicting Three-Dimensional Non-Newtonian Flows in Metering Channels. SPE ANTEC Tech. Pap..

[31] Marschik C., Roland W., Miethlinger J. (2018). A Network-Theory-Based Comparative Study of Melt-Conveying Models in Single-Screw Extrusion: A. Isothermal Flow. Polymers.

[32] Luger H.J., Roland W., Loew-Baselli B., Miethlinger J. (2018). A Network-Analysis-Based Comparative Study of the Throughput Behavior in Double Wave Screw Geometries. SPE ANTEC Tech. Pap..

[33] McKelvey J.M. (1954). Theory of Adiabatic Extruder Operation. Ind. Eng. Chem..

[34] Potente H., Obermann C. (1999). Screw Drive Power of Single Screw Plasticating Units with Smooth Barrels. Int. Polym. Process..

[35] Campbell G.A., Wang C., Cheng H., Bulwinkel M., te-Riele M.A. (2001). Investigation of Flow Rate and Viscous Dissipation in a Single Screw Pump-Extruder. Int. Polym. Process..

[36] Derezinski S.J. (1987). Dimensionless Curves for Extruder Melt Temperature and Flow. J. Plast. Film Sheet..

[37] Derezinski S.J. (1996). Heat Transfer Coefficients in Extruder Melt Sections. SPE ANTEC Tech. Pap..

[38] Derezinski S.J. (2013). Universal Melt Temperature Diagram. SPE ANTEC Tech. Pap..

[39] Roland W., Miethlinger J. (2017). Analyzing the Viscous Dissipation of a Two-Dimensional Flow of Non-Newtonian Fluids in Single-Screw Extruders. SPE ANTEC Tech. Pap..

[40] Roland W., Miethlinger J. (2018). Heuristic Analysis of Viscous Dissipation in Single-Screw Extruders. Polym. Eng. Sci..

[41] Koza J.R. (1992). Genetic Programming: On the Programming of Computers by Means of Natural Selection.

[42] Schmidt M., Lipson H. (2009). Distilling Free-Form Natural Laws from Experimental Data. Science.

[43] Bramerdorfer G., Winkler S.M., Kommenda M., Weidenholzer G., Silber S., Kronberger G., Affenzeller M., Amrhein W. (2014). Using FE Calculations and Data-Based System Identification Techniques to Model the Nonlinear Behavior of PMSMs. IEEE Trans. Ind. Electron..

[44] Kronberger G., Kommenda M., Lughofer E., Saminger-Platz S., Promberger A., Nickel F., Winkler S., Affenzeller M. (2018). Using robust generalized fuzzy modeling and enhanced symbolic regression to model tribological systems. Appl. Soft Comp..

[45] Kronberger G., Fink S., Kommenda M., Affenzeller M., Di Chio C. (2011). Macro-economic Time Series Modeling and Interaction Networks. Applications of Evolutionary Computation.

[46] Sun J., Rauwendaal C. (2002). Analysis of Flow in Single Screw Extruders. SPE ANTEC Tech. Pap..

[47] Roland W., Marschik C., Löw-Baselli B., Miethlinger J. (2018). The Effect of Channel Curvature on the Flow Rate and Viscous Dissipation of Power-Law Fluids. SPE ANTEC Tech. Pap..

[48] Durst F. (2008). Fluid Mechanics: An Introduction to the Theory of Fluid Flows.

[49] Langtangen H.P., Pedersen G.K. (2016). Scaling of Differential Equations.

[50] Wagner S., Kronberger G., Beham A., Kommenda M., Scheibenpflug A., Pitzer E., Vonolefen S., Kofler M., Winkler S., Dorfer V., Klempous R., Nikodem J., Jacak W., Chaczko Z. (2014). Architecture and Design of the HeuristicLab Optimization Environment. Advanced Methods and Applications in Computational Intelligence.

[51] Poli R., Langdon W.B., McPhee N.F. (2008). A Field Guide to Genetic Programming.

[52] Affenzeller M., Wagner S., Winkler S., Beham A. (2018). Genetic Algorithms and Genetic Programming: Modern Concepts and Practical Applications.

[53] Deb K., Pratap A., Agarwal S., Meyarivan T. (2002). A Fast and Elitist Multiobjective Genetic Algorithm: NSGA-II. IEEE Trans. Evol. Comp..

[54] Affenzeller M., Wagner S., Ribeiro B., Albrecht R.F., Dobnikar A., Pearson D.W. (2005). Offspring Selection: A New Self-Adaptive Selection Scheme for Genetic Algorithms. Adaptive and Natural Computing Algorithms.

[55] Kommenda M., Kronberger G., Affenzeller M., Winkler S., Burlacu B., Riolo R., Worzel W., Kotanchek M., Kordon A. (2016). Evolving Simple Symbolic Regression Models by Multi-Objective Genetic Programming. Genetic Programming Theory and Practice XIII. Genetic and Evolutionary Computation.

[56] Schöppner V. (1995). Simulation der Plastifiziereinheit von Einschneckenextrudern. Ph.D. Thesis.

[57] Aigner M. (2004). Computational and Experimental Modelling of Transport Phenomena in Single Screw Plasticating Units under Consideration of the Melt Quality. Ph.D. Thesis.

